# HCC prediction models in chronic hepatitis B patients receiving entecavir or tenofovir: a systematic review and meta-analysis

**DOI:** 10.1186/s12985-023-02145-5

**Published:** 2023-08-15

**Authors:** Xiaolan Xu, Lushun Jiang, Yifan Zeng, Liya Pan, Zhuoqi Lou, Bing Ruan

**Affiliations:** 1 State Key Laboratory for Diagnosis and Treatment of Infectious Diseases, National Clinical Research Center for Infectious Diseases, Collaborative Innovation Center for Diagnosis and Treatment of Infectious Diseases, The First Affiliated Hospital, National Medical Center for Infectious Diseases, Zhejiang University School of Medicine, 79 Qingchun Road, Shangcheng District, Hangzhou, 310000 China; 2 Center for General Practice Medicine, Department of Infectious Diseases, Zhejiang Provincial People’s Hospital (Affiliated People’s Hospital), Hangzhou Medical College, Hangzhou, 310000 China

**Keywords:** Risk factors, Discrimination, Calibration, Predictive value of tests, Surveillance

## Abstract

**Background:**

Our study aimed to compare the predictive performance of different hepatocellular carcinoma (HCC) prediction models in chronic hepatitis B patients receiving entecavir or tenofovir, including discrimination, calibration, negative predictive value (NPV) in low-risk, and proportion of low-risk.

**Methods:**

We conducted a systematic literature research in PubMed, EMbase, the Cochrane Library, and Web of Science before January 13, 2022. The predictive performance was assessed by area under receiver operating characteristic curve (AUROC), calibration index, negative predictive value, and the proportion in low-risk. Subgroup and meta-regression analyses of discrimination and calibration were conducted. Sensitivity analysis was conducted to validate the stability of the results.

**Results:**

We identified ten prediction models in 23 studies. The pooled 3-, 5-, and 10-year AUROC varied from 0.72 to 0.84, 0.74 to 0.83, and 0.76 to 0.86, respectively. REAL-B, AASL-HCC, and HCC-RESCUE achieved the best discrimination. HCC-RESCUE, PAGE-B, and mPAGE-B overestimated HCC development, whereas mREACH-B, AASL-HCC, REAL-B, CAMD, CAGE-B, SAGE-B, and aMAP underestimated it. All models were able to identify people with a low risk of HCC accurately. HCC-RESCUE and aMAP recognized over half of the population as low-risk. Subgroup analysis and sensitivity analysis showed similar results.

**Conclusion:**

Considering the predictive performance of all four aspects, we suggest that HCC-RESCUE was the best model to utilize in clinical practice, especially in primary care and low-income areas. To confirm our findings, further validation studies with the above four components were required.

**Supplementary Information:**

The online version contains supplementary material available at 10.1186/s12985-023-02145-5.

## Background

Chronic hepatitis B virus (HBV) infection is associated with life-threatening liver conditions like hepatocellular carcinoma (HCC) [1]. The early detection of HCC and stratified care of distinct risk populations were critical to minimize the harm of liver complications. Patients could be classified into different risk levels of developing HCC over time with HCC risk prediction models. There were models developed in untreated patients like REACH-B (Risk Estimation for HCC in Chronic Hepatitis B)and NGM-HCC (Nomograms for Risk of Hepatocellular Carcinoma) [2, 3], in mixed patients like CU-HCC (Chinese University-HCC), LSM-HCC (Liver Stiffness Measurement based-HCC), GAG-HCC (Guide with Age, Gender, HBV DNA, Core promoter mutations and Cirrhosis), and aMAP (the Age-Male-ALBI-Platelets Score), and models developed in treated patients [4–6].

Given that antiviral therapy was commonly employed in the present society, models developed in treated patients may have a greater advantage in the accuracy of predictions. The mREACH-B (modified REACH-B), PAGE-B (Platelet, Age, Gender and HBV), mPAGE-B (modified PAGE-B), HCC-RESCUE (HCC-Risk Estimating Score in CHB patients Under Entecavir), CAMD (the Cirrhosis, Age, Male sex, and Diabetes Mellitus Score), AASL-HCC (Age, Albumin, Sex, Liver Cirrhosis-HCC Scoring System), CAGE-B (Cirrhosis and Age Score), SAGE-B (Stiffness and Age Score), and REAL-B (Real-world Effectiveness from the Asia Pacific Rim Liver Consortium for HBV) were initially developed in patients treated with different antiviral drugs [7–15]. It was important to combine information from all derivation and external validation studies for the same model to assess the prediction performance across diverse populations. Furthermore, for some low-risk patients, needless HCC screening and surveillance may result in potential physical, financial, and psychological harms [16]. According to the guideline, HCC surveillance was cost-effective if the annual risk of HCC was ≥ 0.2% per year [17]. As a result, the fraction of low-risk population highlighted by models, as well as the ability to exclude individuals who are unlikely to develop HCC, should be considered.

Presently, entecavir and tenofovir were the first-line medications suggested in the guidelines for antiviral treatment [1, 18, 19]. Thus, our study systematically assessed the prediction performance of the above models in patients treated with entecavir and tenofovir in a meta-analysis.

## Methods

The systematic review and meta-analysis was reported according to the Preferred Reporting Items for Systematic Reviews and Meta-Analyses (PRISMA) and was registered on PROSPERO (ID: CRD42022303167).

### Search strategy

We searched literatures published before January 13, 2022 in PubMed, EMbase, the Cochrane Library, and Web of Science. There were no limits on language or publication dates. Keywords of HCC, CHB, prediction models, et al. were used (Content 1 in Additional file 1). We also looked up references in relevant reviews and original publications to see if there were any studies we had overlooked.

### Selection criteria

HCC prediction models built in treated CHB patients were selected in our study, including mREACH-B, PAGE-B, mPAGE-B, HCC-RESCUE, CAMD, AASL-HCC, CAGE-B, SAGE-B, and REAL-B. Even though aMAP was created in a mixed population, we included it in our study due to the large sample size in the derivation study. Both derivation studies and validation studies were retrieved. Exclusion criteria were as follows: (1) reviews or meta-analyses, (2) conference abstracts, (3) letters, editorials, and case reports, (4) full-text not in English, (5) update models without external validation, (6) validation cohort including untreated patients or patients treated with other oral antiviral medications, (7) insufficient data for analysis. The study focused on 3-, 5-, and 10-year HCC prediction performance. XX and LJ independently examined titles and abstracts, and studies that met the inclusion criteria were retrieved for full-text evaluation. Two independent investigators were also responsible for data extraction (XX and LJ). Any discrepancies were resolved by a third investigator (YZ).

### Data extraction

The following data were extracted from these studies: author, publication year, study type, region, race, setting, recruitment period, sample size, follow-up duration, HCC cases, study interval, type of antiviral treatment received, baseline demographic and medical history (age, sex, proportion of cirrhosis, alcohol abuse, and diabetes), baseline laboratory results (hepatitis B e antigen [HBeAg] status, HBV DNA quantitative, alanine aminotransferase [ALT], platelets, albumin, total bilirubin, alpha-fetoprotein, liver stiffness measurement, and prediction score), area under the receiver operator characteristic curve (AUROC) with 95% confidence interval (CI), observed (O) events and expected (E) events, and negative predictive value (NPV) with 95% CI in the low-risk group. For external validation of different existing models or different cohorts, information was extracted separately.

According to Prediction model Risk of Bias Assessment Tool (PROBAST), which was organized into the following 4 domains: participants, predictors, outcome, and analysis, the quality of the original studies was evaluated.

### Statistical analysis

Both derivation and external validation studies were included in meta-analysis. In the meta-analysis, effect measures included AUROC with 95% CI, total O:E ratio with standard error, NPV with 95% CI, and proportion in low-risk group with 95% CI. The predicted occurrences were estimated by multiplying the cumulative HCC incidence by the number of patients in each risk group. Variance of O:E ratio was calculated according to the equation recommended by Debray et al. [20]. When generating 95% CI for average performance, the random-effects model was used. I^2^ statistics were used to measure between-study heterogeneity. I^2^ statistic > 50% was regarded as moderate heterogeneity and I^2^ statistics > 75% was considered as severe heterogeneity. Subgroup analysis was conducted by cirrhotic status (cirrhotic/non-cirrhotic) and race (Caucasian/Asian) to explore the heterogeneity. Meta-regression analysis adjusted by the Hartung-Knapp method was conducted. Besides, sensitivity analysis by omitting anyone research was conducted to study the impact of individual studies on the average performance. Publication bias was analyzed by funnel plots for models that included ten or more studies [21]. All analyses were performed using Review Manager version 5.4 (Cochrane, London, United Kingdom) and StataMP software version 15 (StataCorp LLC, Texas, USA).

## Results

**Fig. 1:**
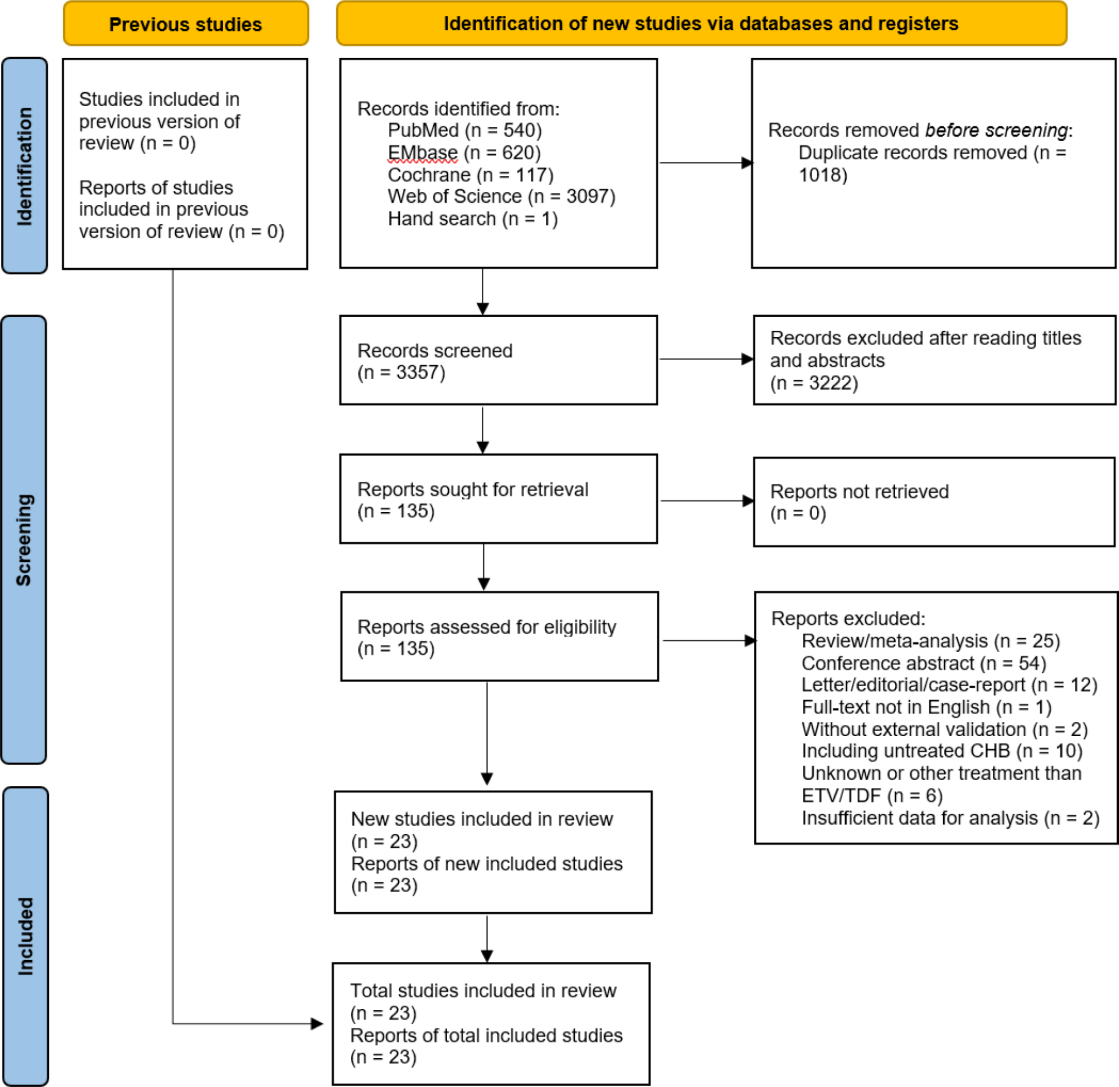
Flow diagram for the systematic analysis and meta-analysis

We identified 23 publications for the systematic review and meta-analysis after screening 4374 studies from four databases, which included 153,445 CHB patients and 5133 HCC cases (Fig. 1). External validation was not performed in the original research for three model derivation investigations (mREACH-B, PAGE-B, CAGE-B, and SAGE-B). External validation studies or derivation and external validation studies of six models made up the remaining research (HCC-RESCUE, CAMD, mPAGE-B, AASL-HCC, REAL-B, and aMAP). PAGE-B and mPAGE-B were the most commonly externally validated in 19 and 14 studies, respectively, whereas REAL-B and mREACH-B were only validated in one study, respectively. Other models were also frequently validated as follows: CAMD (n = 6), HCC-RESCUE (n = 5), AASL-HCC (n = 4,), CAGE-B and SAGE-B (n = 3), and aMAP (n = 2).

### Characteristics of the included studies

The participants were recruited retrospectively using hospital medical records, whereas Hsu et al. and Yip et al. used an insurance database and the Clinical Data Analysis and Reporting System to perform their studies [11, 22]. Different from other studies, Gui et al. compared model performance in cirrhotic patients [23], and Kim et al. studied veterans in United States [24]. Most models were developed in Asian populations, except for PAGE-B, CAGE-B, and SAGE-B, which were derived from Caucasian populations. Except for REAL-B, which was developed in individuals whose treatment regimen included other oral antiviral medicines, most models were developed in patients treated with entecavir or tenofovir. And aMAP was developed in mixed patients with a treatment proportion of 78%. The number of parameters in the models ranged from three to seven. Age and sex were nearly included in all models and other parameters included albumin, total bilirubin, platelets, cirrhosis, liver stiffness measurement, ALT, HBeAg status, diabetes, alcohol abuse, and alpha-fetoprotein (Table 1). The REAL-B and aMAP derivation cohorts were not included in the meta-analysis because their participants did not match the inclusion criteria.

**Table 1:** Summary of hepatitis B virus-hepatocellular carcinoma prediction models in the derivation studies

Model	Region	Race	Follow-up, month	HCC cases /Sample size	Median Age, year	Male, %	Cirrhosis, %	Predictor variables	Cut-offs
mREACH-B Lee [7], 2014	Korea	Asian	43	15/192	49	69.8	46.9	*Age* Sex ALT HBeAg LSM	-
PAGE-B Papatheodoridi [8], 2016	Greece/Italy/Spain/Netherlands/Turkey	Caucasian	44	51/1325	52	70.0	20.0	*Age* Sex Platelets	Low risk: 0–9 Intermedia risk: 10–17 High risk: 18–25
mPAGE-B Kim [9], 2018	Korea	Asian	49	132/2001	50	64.1	19.1	*Age* Sex Platelets Albumin	Low risk: 0–8 Intermedia risk: 9–12 High risk: 13–21
HCC-RESCUE Sohn [10], 2017	Korea	Asian	25	58/990	47	65.0	39.0	*Age* Sex Cirrhosis	Low risk: 18–64 Intermedia risk: 65–84 High risk: 85–113
CAMD Hsu [11], 2018	Taiwan	Asian	26	596/65,426	48	74.0	26.5	Age Sex Diabetes Cirrhosis	Low risk: 0–7 Intermedia risk: 8–13 High risk: 14–19
AASL-HCC Yu [12], 2019	Korea	Asian	49	56/944	50	62.1	39.3	*Age* Sex Albumin Cirrhosis	Low risk: 0–5 Intermedia risk: 6–19 High risk: 2–29
aMAP Fan [15], 2020	China	Asian	43	95/3688	38	80.7	19.3	*Age* Sex *Albumin* *Total bilirubin* *Platelets*	Low risk: 0–50 Intermedia risk: 50–60 High risk: 60–100
CAGE-B Papatheodoridi [13], 2020	Greece/Italy/Spain/Netherlands/Turkey	Caucasian	101	33/1427	52	69.5	25.9	Age at year 5 Baseline cirrhosis LSM at year 5	Low risk: 0–5 Intermedia risk: 6–10 High risk: 11–16
SAGE-B Papatheodoridi [13], 2020	Greece/Italy/Spain/Netherlands/Turkey	Caucasian	101	33/1427	52	69.5	25.9	Age at year 5 LSM at year 5	Low risk: 0–5 Intermedia risk: 6–10 High risk: 11–15
REAL-B Yang [14], 2020	United States/Asia-Pacific region	Asian	29,572 person-years	378/5365	48	69.2	20.2	Male gender *Age* Alcohol Diabetes Cirrhosis Platelets ɑ-fetoprotein	Low risk: 0–3 Intermedia risk: 4–7 High risk: 8–13

The italic indicates a continuous variable and the other indicates a categorical variable. HCC, hepatocellular carcinoma; ALT, alanine aminotransferase; HBeAg, hepatitis B e antigen; LSM, liver stiffness measurement; mREACH-B, Modified Risk Estimation for Hepatocellular Carcinoma in Chronic Hepatitis B; PAGE-B, Platelet, Age, Gender and HBV; mPAGE-B, modified Platelet, Age, Gender and HBV; HCC-RESCUE, HCC-Risk Estimating Score in CHB patients Under Entecavir; CAMD, the Cirrhosis, Age, Male sex, and Diabetes Mellitus Score; AASL-HCC, Age, Albumin, Aex, Liver Cirrhosis-HCC scoring system; aMAP: the Age-Male-ALBI-Platelets Score; CAGE-B, Cirrhosis and Age Score; SAGE-B, Stiffness and Age Score; REAL-B, Real-world Effectiveness from the Asia Pacific Rim Liver Consortium for HBV.

### Risk of bias and applicability assessment

The details of the risk of bias and applicability were depicted in Table S1-2 and Figure S1-2. According to PROBAST, the predictors and outcome had a low risk of bias, but the participants and analysis had a high risk of bias in 17.4% and 52.1% of studies, respectively. In terms of analysis, model calibration was not performed in eight studies (34.8%) and four studies (17.4%) had a small number of HCC cases. Except for 17.4% of research, which had a high risk of participants, most models had a low risk of applicability.

**Fig. 2:**
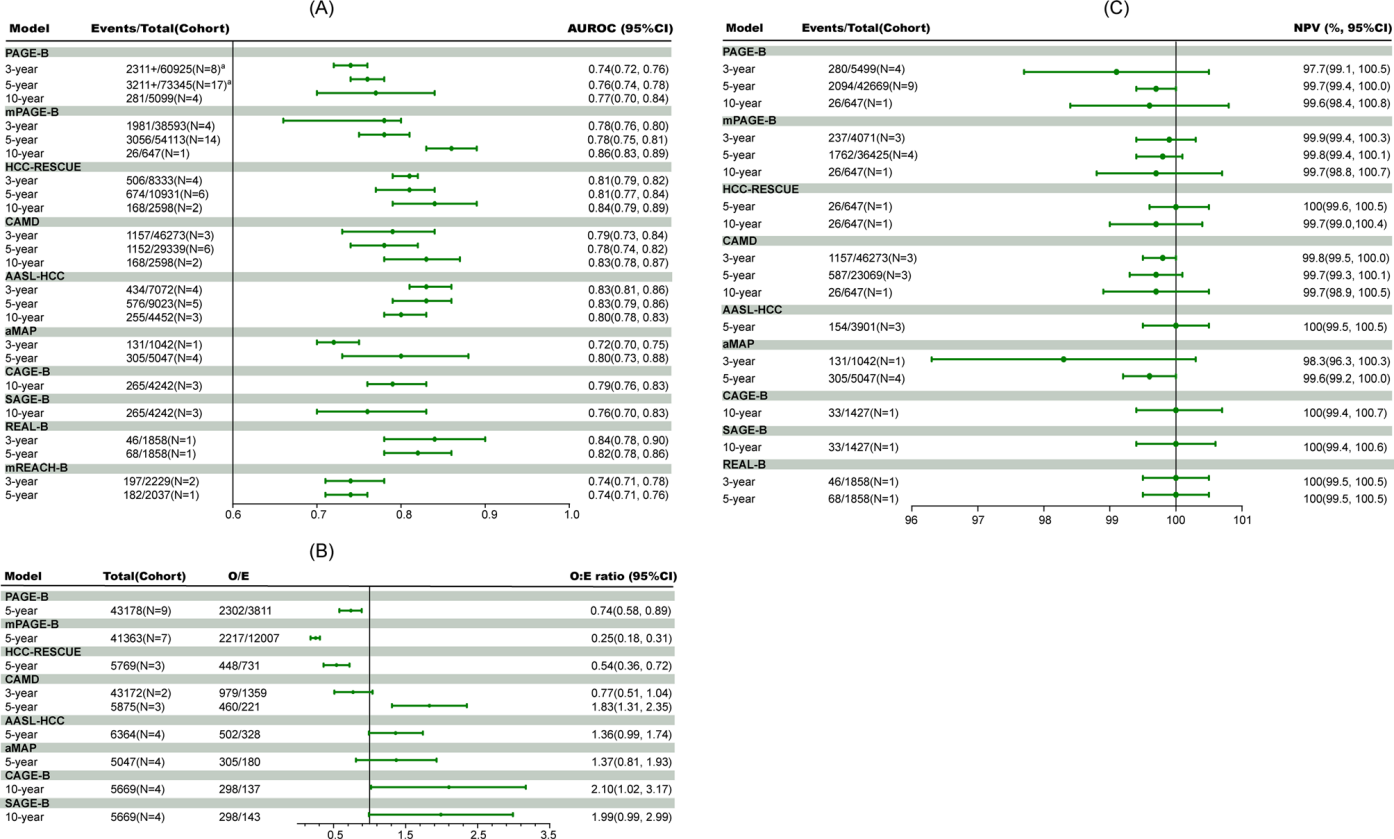
The discrimination (**A**), calibration (**B**) performance and negative predictive values in the low-risk group (**C**) of HCC prediction models in meta-analysis. ^a^HCC events were not reported by Hsu et al. [11], which included 17,984 participants in the study. AUROC, area under the receiver operator characteristic curve; CI, confidence interval; O:E ratio, observed events versus expected events ratio; NPV, negative predictive value; HCC, hepatocellular carcinoma; mREACH-B, Modified Risk Estimation for Hepatocellular Carcinoma in Chronic Hepatitis B; PAGE-B, Platelet, Age, Gender and HBV; mPAGE-B, modified Platelet, Age, Gender and HBV; HCC-RESCUE, HCC-Risk Estimating Score in CHB patients Under Entecavir; CAMD, the Cirrhosis, Age, Male sex, and Diabetes Mellitus Score; AASL-HCC, Age, Albumin, Aex, Liver Cirrhosis-HCC scoring system; aMAP: the Age-Male-ALBI-Platelets Score; CAGE-B, Cirrhosis and Age Score; SAGE-B, Stiffness and Age Score; REAL-B, Real-world Effectiveness from the Asia Pacific Rim Liver Consortium for HBV

### Meta-analysis

The characteristics of included studies in meta-analysis were shown in Table 2. The pooled 3-, 5-, and 10-year AUROC varied from 0.72 to 0.84 (aMAP: 0.72, 95% CI 0.70–0.75; REAL-B: 0.84, 95% CI 0.78–0.90), 0.74 to 0.83 (mREACH-B: 0.74, 95% CI 0.71–0.76; AASL-HCC: 0.83, 95% CI 0.79–0.86), and 0.76 to 0.86 (SAGE-B: 0.76, 95% CI 0.70–0.83; mPAGE-B: 0.86, 95% CI 0.83–0.89), respectively (Fig. 2, Figure S3, Table S3). When predicting 3-year HCC incidence, REAL-B, AASL-HCC, and HCC-RESCUE models had better discrimination with an AUROC > 0.80, while mREACH-B, PAGE-B and aMAP showed an AUROC < 0.75. When predicting 5-year HCC incidence, AASL-HCC, REAL-B, HCC-RESCUE, and aMAP models performed better with an AUROC ≥ 0.80, followed by mPAGE-B, CAMD, and PAGE-B with an AUROC > 0.75, while mREACH-B showed an AUROC < 0.75. When predicting 10-year HCC incidence, with an AUROC ≥ 0.80, mPAGE-B, HCC-RESCUE, CAMD, and AASL-HCC outperformed CAGE-B, PAGE-B, and SAGE-B (> 0.75).

**Table 2:** Characteristics of derivation and external validation cohorts included in the meta-analysis

No.	Study	Region	Race	Model	Study setting	Recruitment period	Follow-up (month)	HCC cases /Sample size
1	Lee [7], 2014	Korea	Asian	mREACH-B	Hospital	2007–2011	43	15/192
2	Papatheodoridis [8], 2016	Greece/Italy/Spain/Netherlands/Turkey	Caucasian	PAGE-B	Hospital	NA	44	51/1325
3	Chen [34], 2017	China	Asian	PAGE-B	Hospital	2007–2012	NA	105/803
4	Kim [35], 2017	Korea	Asian	PAGE-B	Hospital	2006–2015	44	36/1092
5	Sohn [10], 2017	Korea	Asian	HCC-RESCUE	Hospital	NA	42	85/1071
6	Hsu [11], 2018	Hong Kong	Asian	CAMD/PAGE-B	Insurance database	2004–2016	33	478/19321
7 − 1	Kim [9], 2018	Korea	Asian	PAGE-B	Hospital	2007–2016	49	132/2001
7 − 2	Kim, 2018	Korea	Asian	PAGE-B/mPAGE-B	Hospital	2007–2016	49	72/1000
8	Yu [12], 2019	Korea	Asian	AASL-HCC	Hospital	2007–2017	41	24/298
9 − 1	Fan [15], 2020	Greece/Italy/Spain/ Netherlands/Turkey	Caucasian	aMAP/PAGE-B	Hospital	NA	91	139/1938
9 − 2	Fan, 2020	North America/Europe /the Asian-Pacific region	Asian	aMAP/PAGE-B/mPAGE-B	Hospital	2005–2006	55	27/1495
9 − 3	Fan, 2020	North America/Europe /the Asian-Pacific region	Caucasian	aMAP/PAGE-B/mPAGE-B	Hospital	2013–2014	63	8/572
10	Kim [25], 2020	Korea	Asian	CAMD/PAGE-B/mPAGE-B	Hospital	2009–2014	58	292/3277
11	Kirino [36], 2020	Japan	Asian	PAGE-B/mPAGE-B	Hospital	2006–2018	61	33/443
12	Papatheodoridis [13], 2020	Greece/Italy/Spain/Netherlands/Turkey	Caucasian	CAGE-B/SAGE-B	Hospital	NA	101	33/1427
13	Yip [22], 2020	Hong Kong	Asian	PAGE-B/mPAGE-B	CDARS	2005–2018	47	1532/32150
14	Ahn [37], 2021	Korea	Asian	PAGE-B/mPAGE-B	Clinical trial	2012–2015	66	96/686
15	Chang [38], 2021	Korea	Asian	AASL-HCC/HCC-RESCUE/PAGE-B/mPAGE-B	Hospital	2007–2014	58	280/3171
16 − 1	Chon [39], 2021	Korea	Asian	PAGE-B/mPAGE-B	Hospital	2007–2017	43	117/1211
16 − 2	Chon, 2021	Korea	Asian	PAGE-B/mPAGE-B	Hospital	2007–2017	43	42/973
17	Gui [23], 2021	China	Asian	aMAP/CAMD/PAGE-B/mPAGE-B	Hospital	2005–2018	41	131/1042
18	Güzelbulut [30], 2021	Turkey	Caucasian	HCC-RESCUE/CAMD/PAGE-B/mPAGE-B	Hospital	2007–2018	47	26/647
19	Lee [41], 2021	Korea	Asian	PAGE-B/mPAGE-B/mREACH-B/mREACH-B	Hospital	2007–2018	58	182/2037
20	Lim [42], 2021	Korea	Asian	CAGE-B/SAGE-B/AASL-HCC/PAGE-B/mPAGE-B	Hospital	2009–2015	93	57/1557
21	Papatheodoridi [43], 2021	Greece/Italy/Spain/ Netherlands/Turkey	Caucasian	PAGE-B/HCC-RESCUE/CAMD/mPAGE-B/AASL-HCC/CAGE-B/SAGE-B	Hospital	NA	91	142/1951
22	Chon [40], 2022	Korea	Asian	CAGE-B/SAGE-B	Hospital	2006–2011	118	66/734
23	Kim [24], 2022	United States	White/Black/Asian/Other	PAGE-B/mPAGE-B/HCC-RESCUE/CAMD/REAL-B/AASL-HCC	Veterans Administration	2008–2017	59	83/3101
No.	Study	Age	Male (%)	Cirrhosis (%)	Alcohol (%)	Diabetes (%)	Treatment (naïve/experienced)	HBeAg positive (%)
1	Lee, 2014	49	69.8	46.9	26.0	6.3	ETV (NA)	52.1
2	Papatheodoridis, 2016	52	70.0	20.0	NA	NA	ETV/TDF (NA)	NA
3	Chen, 2017	50 ± 17	71.9	35.9	NA	11.2	ETV (naïve)	35.2
4	Kim, 2017	48 ± 12	61.2	36.5	NA	NA	ETV/TDF (naïve/experienced)	56.2
5	Sohn, 2017	47 ± 12	63.0	35.0	NA	NA	ETV (naïve)	61.0
6	Hsu, 2018	52 [41, 60]	66.1	7.1	NA	16.0	ETV/TDF (naïve)	NA
7 − 1	Kim, 2018	50 [42, 57]	64.1	19.1	NA	NA	ETV/TDF (naïve/experienced)	33.9
7 − 2	Kim, 2018	50 [42, 56]	63.1	20.1	NA	NA	ETV/TDF (naïve/experienced)	34.5
8	Yu, 2019	53 [43, 60]	58.7	38.9	NA	NA	ETV/TDF (naïve)	65.4
9 − 1	Fan, 2020	54 [44, 63]	70.6	27.4	NA	NA	ETV/TDF (naïve/experienced)	18.0
9 − 2	Fan, 2020	40 [32, 48]	65.4	11.4	NA	NA	TDF (experienced)	63.5
9 − 3	Fan, 2020	38 [28, 48]	77.4	17.6	NA	NA	TDF/TAF (experienced)	46.2
10	Kim, 2020	49 ± 12	62.6	32.4	NA	8.7	ETV/TDF (naïve)	NA
11	Kirino, 2020	51 ± 13	63.0	NA	NA	NA	ETV/TDF/TAF (naïve/experienced)	41.0
12	Papatheodoridis, 2020	52	69.5	25.9	14.7	8.2	ETV/TDF (naïve/experienced)	18.4
13	Yip, 2020	53 ± 13	64.9	14.4	2.0	23.0	ETV/TDF (naïve/experienced)	NA
14	Ahn, 2021	47 ± 11	62.8	40.3	NA	NA	ETV/TDF (naïve)	39.7
15	Chang, 2021	49 ± 12	62.3	32.8	NA	8.8	ETV/TDF (naïve)	49.4
16 − 1	Chon, 2021	50 ± 11	59.8	45.9	NA	15.9	ETV/TDF (naïve)	49.9
16 − 2	Chon, 2021	47 ± 11	60.3	40.6	NA	8.7	ETV/TDF (naïve)	60.6
17	Gui, 2021	48 ± 12	67.3	100.0	NA	8.2	ETV (naïve)	42.1
18	Güzelbulut, 2021	45 ± 14	64.9	27.8	NA	15.0	ETV/TDF (naïve)	24.0
19	Lee, 2021	50 [41, 57]	57.9	49.9	NA	NA	ETV/TDF (naïve)	50.3
20	Lim, 2021	47 ± 11	63.8	27.7	NA	NA	ETV/TDF (NA)	60.5
21	Papatheodoridis, 2021	53 ± 14	71.0	27.0	19.6	9.0	ETV/TDF (NA)	18.0
22	Chon, 2022	48 ± 40	55.0	47.3	NA	4.8	ETV (naïve)	53.4
23	Kim, 2022	57 ± 13	94.9	32.2	30.2	26.9	ETV/TDF (naïve)	42.5
No.	Study	HBV DNA, log_10_IU/ml	ALT, IU/l	Platelets, 10^3^/mm^3^	Albumin, g/dL	Total bilirubin, mg/dl	ɑ-fetoprotein, ng/ml	LSM, kPa
1	Lee, 2014	0	26	NA	NA	0.9	3	8.8
2	Papatheodoridis, 2016	NA	NA	191	NA	NA	NA	NA
3	Chen, 2017	6.0	106	163	4.1	1.0	6.2	NA
4	Kim, 2017	5.7	238	162	4.2	0.9	NA	NA
5	Sohn, 2017	6.6	234	162	3.9	NA	NA	NA
6	Hsu, 2018	NA	NA	NA	NA	NA	NA	NA
7 − 1	Kim, 2018	3.0	57	158	4.2	0.7	NA	NA
7 − 2	Kim, 2018	3.0	54	161	4.2	1.0	NA	NA
8	Yu, 2019	6.8	89	154	3.7	1.0	4.3	NA
9 − 1	Fan, 2020	5.6	43	187	4.4	12.0	NA	NA
9 − 2	Fan, 2020	7.2	84	191	4.3	10.3	NA	NA
9 − 3	Fan, 2020	7.2	103	201	4.3	10.3	NA	NA
10	Kim, 2020	NA	NA	166	4.1	1.0	NA	NA
11	Kirino, 2020	6.4	42	170	4.2	0.7	3.7	NA
12	Papatheodoridis, 2020	NA	NA	194	NA	NA	NA	NA
13	Yip, 2020	NA	56	183	4.1	19.4	NA	NA
14	Ahn, 2021	11.0	199	161	4	1.3	37.9	NA
15	Chang, 2021	5.7	97	166	4.0	1.1	34.2	NA
16 − 1	Chon, 2021	NA	52	156	4.0	0.9	NA	16.0
16 − 2	Chon, 2021	NA	89	163	4.1	0.9	NA	14.7
17	Gui, 2021	5.1	83	113	4.0	24.4	NA	NA
18	Güzelbulut, 2021	6.0	106	193	4.0	1.2	7.2	NA
19	Lee, 2021	NA	48	168	4.2	0.8	4.0	7.6
20	Lim, 2021	5.8	57	166	4.2	0.9	NA	7.4
21	Papatheodoridis, 2021	NA	NA	191	NA	NA	NA	NA
22	Chon, 2022	6.6	87	157	NA	0.9	NA	13.2
23	Kim, 2022	NA	101	191	3.8	1.2	9.3	NA

HCC, hepatocellular carcinoma; ALT, alanine aminotransferase; HBeAg, hepatitis B e antigen; LSM, liver stiffness measurement; ETV, entecavir; TDF, tenofovir alafenamide; TAF, tenofovir disoproxil fumarate; mREACH-B, Modified Risk Estimation for Hepatocellular Carcinoma in Chronic Hepatitis B; PAGE-B, Platelet, Age, Gender and HBV; mPAGE-B, modified Platelet, Age, Gender and HBV; HCC-RESCUE, HCC-Risk Estimating Score in CHB patients Under Entecavir; CAMD, the Cirrhosis, Age, Male sex, and Diabetes Mellitus Score; AASL-HCC, Age, Albumin, Aex, Liver Cirrhosis-HCC scoring system; aMAP: the Age-Male-ALBI-Platelets Score; CAGE-B, Cirrhosis and Age Score; SAGE-B, Stiffness and Age Score; REAL-B, Real-world Effectiveness from the Asia Pacific Rim Liver Consortium for HBV; CDARS, Clinical Data Analysis and Reporting System.

The pooled 5- and 10-year total O:E ratio ranged from 0.25 to 1.83 (mPAGE-B: 0.25, 95% CI 0.18–0.31; CAMD: 1.83, 95% CI 1.31–2.35) and 1.99 to 2.10 (SAGE-B: 1.99, 95% CI 0.99–2.99; CAGE-B: 2.10, 95%CI 1.02–3.17), respectively (Fig. 2, Figure S4). The pooled 3-year total O:E ratio of CAMD was 0.77 (95% CI 0.51–1.04). HCC-RESCUE, PAGE-B, and mPAGE-B exhibited an overestimation of HCC development, while AASL-HCC, aMAP, CAMD, CAGE-B, and SAGE-B exhibited an underestimation of HCC development.

The pooled 3-, 5-, and 10-year NPVs ranged from 98.3 to 100% (aMAP: 98.3%, 95% CI 96.3-100.3%; REAL-B: 100%, 95% CI 99.5-100.5%), 99.58–100% (aMAP: 99.6%, 95% CI 99.2–100.0%; AASL-HCC: 100%, 95% CI 99.5-100.5%; HCC-RESCUE: 100%, 95% CI 99.6-100.5%; REAL-B: 100%, 95%CI 99.5-100.5%), and 99.6–100% (PAGE-B: 99.6%, 95% CI 98.4-100.8%; CAGE-B: 100%, 95% CI 99.4-100.7%; SAGE-B: 100%, 95%CI 99.4-100.6%), respectively (Fig. 2, Table S4). All models had a high NPV over 99.5% except for aMAP. The proportion of low-risk population ranged from 14.4 to 53.0% (CAGE-B: 14.4%, 95% CI 12.9–16.0%; aMAP: 53.0%, 95% CI 28.5–77.6%) (Table 3). More than half of the population was identified as low-risk by HCC-RESCUE and aMAP.

**Table 3:** The proportion of low-risk population classified by the models in meta-analysis

Model	Sample size	Low-risk proportion, %	95% CI	I^2^	*P*
PAGE-B	45,241 (N = 10)	20.1	16.3, 23.9	98.4%	< 0.001
mPAGE-B	38,997 (N = 5)	26.6	20.2, 33.0	99.0%	< 0.001
HCC-RESCUE	3818 (N = 2)	52.4	50.8, 54.0	0.0%	-
CAMD	50,197 (N = 5)	36.8	30.4, 43.3	99.5%	< 0.001
AASL-HCC	7072 (N = 4)	21.8	12.1, 31.5	99.0%	< 0.001
aMAP	4005 (N = 3)	53.0	28.5, 77.6	99.7%	< 0.001
CAGE-B	1951 (N = 1)	14.4	12.9, 16.0	-	-
SAGE-B	1951 (N = 1)	15.8	14.2, 17.5	-	-
REAL-B	1858 (N = 1)	19.1	17.3, 21.0	-	-

CI, confidence interval; PAGE-B, Platelet, Age, Gender and HBV; mPAGE-B, modified Platelet, Age, Gender and HBV; HCC-RESCUE, HCC-Risk Estimating Score in CHB patients Under Entecavir; CAMD, the Cirrhosis, Age, Male sex, and Diabetes Mellitus Score; AASL-HCC, Age, Albumin, Aex, Liver Cirrhosis-HCC scoring system; aMAP: the Age-Male-ALBI-Platelets Score; CAGE-B, Cirrhosis and Age Score; SAGE-B, Stiffness and Age Score; REAL-B, Real-world Effectiveness from the Asia Pacific Rim Liver Consortium for HBV

### Subgroup analysis and meta-regression

The results of subgroup analysis for discrimination and calibration were presented in Table S5. Only three researches compared the performance of PAGE-B, mPAGE-B, and aMAP in cirrhotic and non-cirrhotic individuals. The discrimination performance was generally better in non-cirrhotic patients than cirrhotic patients. PAGE-B, mPAGE-B, HCC-RESCUE, CAMD, and aMAP exhibited greater AUROC in Caucasians, whereas AASL-HCC, CAGE-B, and SAGE-B had comparable discrimination performance in Asians and Caucasians. Several articles reported the model calibration performance, but the difference in cirrhotic and non-cirrhotic population was not reported. The calibration of the subgroup analysis by race was same as that in meta-analysis. And the underestimation of CAMD seems to be more pronounced in Asians than in Caucasians (O:E ratio 2.38 vs. 1.55). We did a meta-regression analysis for model discrimination and calibration performance and found the heterogeneity could not be explained by race (Figure S5).

### Publication bias and sensitivity analysis

**Fig. 3:**
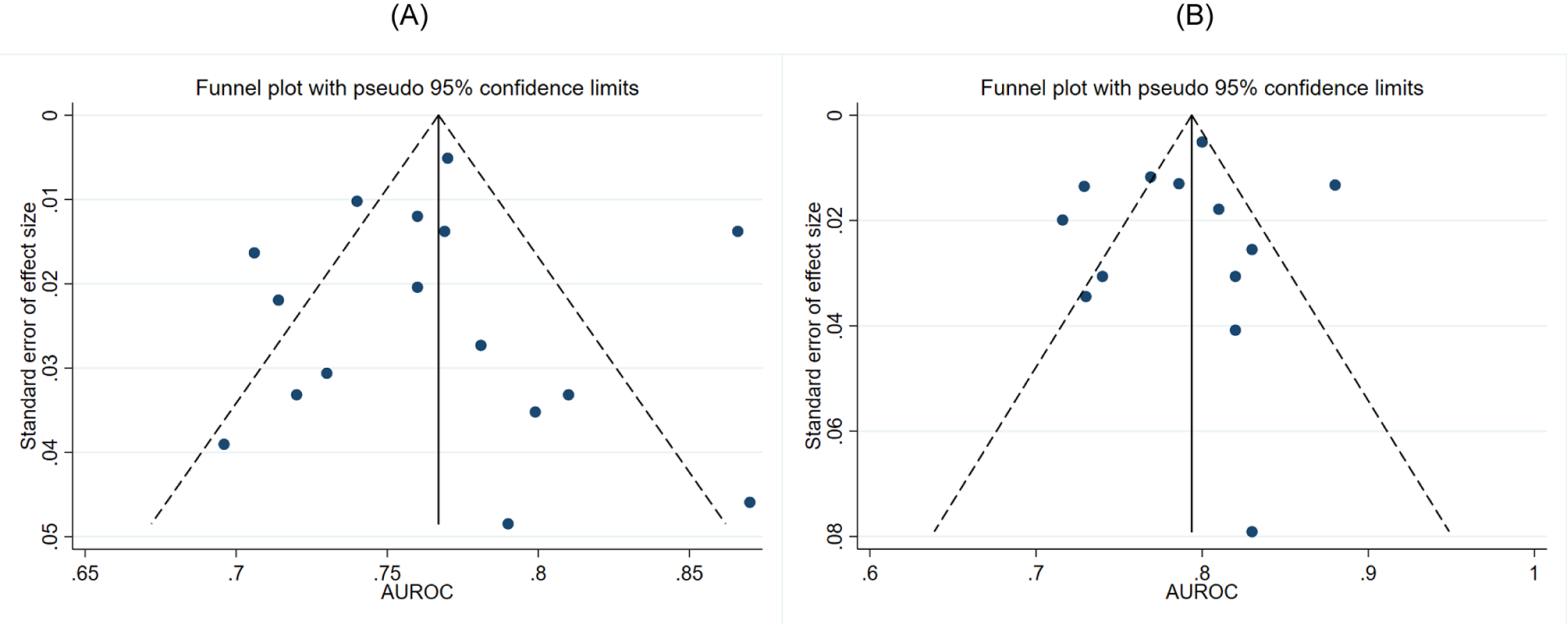
Funnel plot with pseudo 95% confidence limits of 5-year AUROC of PAGE-B (A) and mPAGE-B (B) PAGE-B, Platelet, Age, Gender and HBV; mPAGE-B, modified Platelet, Age, Gender and HBV; AUROC, area under the receiver operator characteristic curve

The funnel plots for the PAGE-B and mPAGE-B model on 5-year discrimination performance were symmetric visually (Fig. 3). Funnel Plots for other models were not analyzed because the number of included studies was small. We mainly discussed the 5- and 10-year predictive performance of model discrimination and calibration, NPV in low-risk, and proportion of low-risk. External validation investigations of REAL-B and mREACH-B were insufficient for sensitivity analysis. After excluding any one research, the pooled 5- or 10-year AUROC of PAGE-B, mPAGE-B, HCC-RESCUE, CAMD, AASL-HCC, CAGE-B, SAGE-B, and aMAP did not change considerably, as shown in Figure S6-7. Sensitivity analysis of calibration was shown in Figure S8-9, and variations in 5-year O:E ratio prediction of CAMD were evident in studies by Hsu and Kim [11, 25]. In Yip et al’s 5-year NPV estimate [22], there was a clear variance in PAGE-B and mPAGE-B (Figure S10). The proportion of low-risk patients detected by AASL-HCC, aMAP, CAMD, PAGE-B, and mPAGE-B did not change significantly in the sensitivity analysis (Figure S11).

### Pair-wise comparison between HCC-RESCUE and other models

**Fig. 4:**
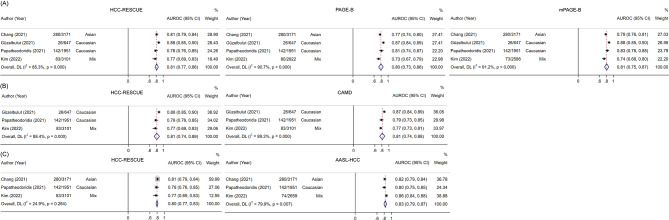
The pair-wise comparison of 5-year AUROC between HCC-RESCUE and other models within the same investigations. (**A**) HCC-RESCUE, PAGE-B, and mPAGE-B; (**B**) HCC-RESCUE and CAMD; (**C**) HCC-RESCUE and AASL-HCC. AUROC, area under the receiver operator characteristic curve; CI, confidence interval; PAGE-B, Platelet, Age, Gender and HBV; mPAGE-B, modified Platelet, Age, Gender and HBV; HCC-RESCUE, HCC-Risk Estimating Score in CHB patients Under Entecavir; CAMD, the Cirrhosis, Age, Male sex, and Diabetes Mellitus Score; AASL-HCC, Age, Albumin, Aex, Liver Cirrhosis-HCC scoring system

We further explored the meta-values of HCC-RESCUE and other models within the same investigations. Only 4 studies have compared the predictive performance of HCC-RESCUE with PAGE-B, mPAGE-B, CAMD, or AASL-HCC. As depicted in Fig. 4, the 5-year AUROC were 0.81 (95% CI 0.77–0.86), 0.80 (95% CI 0.73–0.86), 0.81 (95% CI 0.75–0.87) for HCC-RESCUE, PAGE-B, and mPAGE-B, respectively. The discrimination was also similar between HCC-RESCUE/CAMD (0.81 vs. 0.81) and HCC-RESCUE/AASL-HCC (0.81 vs. 0.83). The proportion of low-risk patients detected by HCC-RESCUE was significantly higher than that by PAGE-B or mPAGE-B (52.4% vs. 23.3% vs. 30%, Table S6).

## Discussion

We conducted a systematic review and meta-analysis of ten HCC prediction models in CHB patients receiving entecavir or tenofovir and compared their predictive performance of discrimination, calibration, NPV in low-risk, and proportion of low-risk. Overall, all models were able to generate satisfactory discrimination with an AUROC > 0.70. In terms of discrimination, calibration, and the capacity to stratify low-risk populations, HCC-RESCUE performed admirably. Different from the previous researches, we also studied the proportions of low-risk in each model and the accuracy of excluding HCC development in low-risk population.

The models included three to seven parameters including age, sex, albumin, total bilirubin, platelets, cirrhosis, liver stiffness measurement, ALT, HBeAg status, diabetes, alcohol abuse, or alpha-fetoprotein. None of the models incorporated viral-related parameters, except for mREACH-B, which included HBeAg status. However, HBV DNA level or HBeAg status were key determinants in models drawn from untreated population or mixed population (CU-HCC, GAG-HCC, LSM-HCC, NGM-HCC) [3–6]. The difference could be explained by that the antiviral therapy was effective in suppressing virus activity. During the 12-month treatment with entecavir or tenofovir, HBV DNA was undetectable in 80% and 69% of patients, respectively, according to a randomized controlled experiment [26]. HBeAg seroconversion rate was over 40% in patients who received 5-year tenofovir or entecavir treatment [27]. According to Papatheodoridis et al., Caucasian patients receiving long-term entecavir or tenofovir had an 8-year survival rate comparable to the general population if HCC had not developed [28]. Thus, viral-related factors might have little effect in predicting long-term HCC development in antiviral-treated patients. We considered these models developed in treated patients were more suitable to predict HCC incidence in the antiviral era.

According to PROBAST, the participant and analysis were the main sources of the bias. Kim et al. compared performance of different models in veterans, which could lead to a significant risk in participant selection [29]. Gui et al. verified the model performance in patients with CHB-related cirrhosis without considering those who did not develop cirrhosis [23]. While Yip and Güzelbulut et al. included decompensated cirrhosis in their validation cohorts, and we figured that model parameters would be unstable in decompensated cirrhosis, thus increasing the models’ inaccuracy [22, 30]. The bias in the analysis was mostly caused by the limited sample size, which resulted in an unreasonable number of HCC instances, as well as the fact that model performance was evaluated inappropriately due to a lack of calibration evaluation.

Overall, REAL-B, AASL-HCC, and HCC-RESCUE models had the best discrimination performance with an AUROC > 0.8. Interestingly, age, sex, and cirrhosis were all included in the above models. HCC was found to be six times more common in cirrhotic patients than in people without cirrhosis, and males were more likely to acquire HCC than females [31]. Also, the risk of developing HCC increased with age. This may indicate that age, sex, and cirrhosis-based models were more accurate in predicting HCC incidence in treated individuals. Similarly, the CAMD model, which included age, sex, diabetes, and cirrhosis, also performed well. Our findings were consistent with a prior meta-analysis that indicated REAL-B and CAMD had the best discrimination performance, in which HCC-RESCUE was not been investigated and AASL-HCC was been validated in one study [32]. Subgroup analysis showed discrimination of aMAP, PAGE-B, and mPAGE-B was better in non-cirrhotic than cirrhotic patients, which has been reported by Wu et al. [32]. However, discrimination evaluation according to cirrhotic status was not available in other model studies. Besides, there was a tendency that discrimination was better in Caucasians than Asians no matter the model was developed in Asian or Caucasian population. The reason for such a racial disparity was currently unknown. To be mentioned, mREACH-B, aMAP and REAL-B were not widely validated in patients treated with entecavir or tenofovir, further studies on these models were needed to verify our findings. Besides, predictive performance in different subgroups should be considered in further validation.

Regarding model calibration performance, HCC-RESCUE, PAGE-B, and mPAGE-B overestimated HCC development, while AASL-HCC, aMAP, CAMD, CAGE-B, and SAGE-B underestimated HCC development. Calibration was not available in REAL-B and mREACH-B. In the previous study, PAGE-B and mPAGE-B had an overestimation which was the same as our findings, while CAMD has a slight overestimation in 3-year period [32]. To classifying patients with high risk of HCC, we figured that the overestimation was preferable than underestimation. Although overestimation may cause excessive surveillance and financial waste, underestimation would lead to the omission of possible HCC patients, putting patients’ lives in jeopardy. Nevertheless, model calibration was only done in two-thirds of the studies involved. As reported in a previous meta-analysis of HCC prediction models, publication compliance with TRIPOD was 74% [33]. Following model validation studies should pay more attention to the completeness of the article according to transparent reporting of individual prognosis or diagnostic multivariate predictive model (TRIPOD) statement.

All models exhibited a high NPV over 99% in low-risk population, indicating the ability of excluding HCC development was admirable. In the 5- or 10-year study period, almost none of the low-risk patients got HCC. Therefore, intensive supervision was not necessary for these patients, potentially reducing the risk of physical, financial, and psychological harms [16]. To our knowledge, the risk stratification proportions were occasionally reported in separate studies and had not been systematically investigated by meta-analysis. To some extent, the more patients were designated as low-risk, the more medical resources could be saved. According to our findings, HCC-RESCUE and aMAP classified over half of the population as low-risk, following by CAMD and mPAGE-B with 36.8% and 26.6%, respectively, while AASL-HCC, PAGE-B, REAL-B, CAGE-B, and SAGE-B was approximately 20%. Thus, HCC-RESCUE made more patients can be spared from HCC screening and save resources, which was especially useful in primary care and low-income areas.

Our research assessed the predictive ability of ten models at multiple time points, and included subgroup analysis based on race and cirrhosis status. We also ran a sensitivity analysis to verify that the results were reliable. The between-study heterogeneity could be partly explained by race and cirrhotic status. And the results were relatively robust in sensitivity analysis. We also used NPV to assess the accuracy of identifying individuals who would not develop HCC in a given time, and we focused on the low-risk proportion divided by each model, which had never been systematically examined before. We proposed that these two characteristics be investigated in model studies since they could play a key role in allocating HCC screening resources.

However, there were several limitations in our study. First, there were insufficient validation cohorts for mREACH-B, aMAP, and REAL-B, because the mREACH-B derivation study did not illustrate the details of the model, and the latter two were newly developed [7, 14, 15]. Second, model calibration, proportion of each risk group, and NPV in low-risk populations were not depicted in every study, which caused that some models (mREACH and REAL-B) were not analyzed for these performance in the meta-analysis and the number of studies was insufficient for some models (HCC-RESCUE, REAL-B, CAGE-B, and SAGE-B). Besides, over half of the studies had a high risk of bias for participant selection or data analysis, but sensitivity analysis showed that our findings remained stable. Finally, the subgroup analysis of cirrhosis status was incomplete because the difference between cirrhotic and non-cirrhotic patients were not displayed in most cohorts. For the similar reason, the discrimination and calibration results could not be stratified according to treatment received. Further external validation studies with more complete information were needed to confirm our findings.

## Conclusion and implications

REAL-B, AASL-HCC, and HCC-RESCUE performed the best discrimination in the meta-analysis of the ten prediction models, although more validation studies of model REAL-B are needed to confirm our findings. AASL-HCC, aMAP, CAMD, CAGE-B, and SAGE-B underestimated HCC development, whereas HCC-RESCUE, PAGE-B, and mPAGE-B overestimated it. Model calibration, proportion of low-risk group, and NPVs were insufficiently reported in many researches, which should be addressed in future model derivation or validation studies. In comparison to other models, HCC-RESCUE identified the most people as low-risk, with a high NPV, indicating that it might be the most appropriate model to be used in primary clinical practice for HCC surveillance.

List of abbreviations: HBV, hepatitis B virus; HCC, hepatocellular carcinoma; CHB, chronic hepatitis B; mREACH-B, modified Risk Estimation for HCC in Chronic Hepatitis B; PAGE-B, Platelet, Age, Gender and HBV; mPAGE-B, modified PAGE-B; HCC-RESCUE, HCC-Risk Estimating Score in CHB patients Under Entecavir; CAMD, the Cirrhosis, Age, Male sex, and Diabetes Mellitus Score; AASL-HCC, Age, Albumin, Sex, Liver Cirrhosis-HCC Scoring System; CAGE-B, Cirrhosis and Age Score; SAGE-B, Stiffness and Age Score; REAL-B, Real-world Effectiveness from the Asia Pacific Rim Liver Consortium for HBV; aMAP, the Age-Male-ALBI-Platelets Score; HBeAg, hepatitis B e antigen; ALT, alanine aminotransferase; AUROC, area under the receiver operator characteristic curve; CI, confidence interval; O:E ratio, observed events versus expected events ratio; NPV, negative predictive value.

### Electronic supplementary material

Below is the link to the electronic supplementary material.

Supplementary Material 1

## Data Availability

All data generated or analyzed during this study are included in this published article [and its supplementary information files].
