# Supplementary material for: HCC prediction models in chronic hepatitis B patients receiving entecavir or tenofovir: a systematic review and meta-analysis
Source: Virol J. 2023 Aug 15;20:180. doi: 10.1186/s12985-023-02145-5 (PMC10428529; doi:10.1186/s12985-023-02145-5)
Supplement: Supplementary file 1 — Supplementary Material 1 [file 12985_2023_2145_MOESM1_ESM.docx]

Supplementary materials

**Content 1** Search strategy in each database

**Content 2** Table S1 Risk of bias and assessment of included studies according to PROBAST

**Content 3** Table S2 Risk of bias and applicability analysis of included studies according to PROBAST

**Content 4** Figure S1 Risk of bias and applicability concerns graph: review authors' judgements about each domain presented as percentages across included studies

**Content 5** Figure S2 Risk of bias and applicability concerns summary: review authors' judgements about each domain for each included study

**Content 6** Figure S3 The 5-year discrimination performance of each HCC prediction models in meta-analysis

**Content 7** Table S3 The 3- and 10-year discrimination performance of HCC prediction models in meta-analysis

**Content 8** Figure S4 The 5- and 10-year calibration performance of each HCC prediction models in meta-analysis

**Content 9** Table S4 The 3-, 5- and 10-year NPV prediction of HCC prediction models in meta-analysis

**Content 10** Table S5 Subgroup analysis of model discrimination (A) and calibration (B) performance

**Content 11** Figure S5 Meta-regression analysis of model discrimination (A) and calibration (B) performance

**Content 12** Figure S6 Sensitivity analysis of 5-year discrimination performance of HCC prediction models in meta-analysis

**Content 13** Figure S7 Sensitivity analysis of 10-year discrimination performance of HCC prediction models in meta-analysis

**Content 14** Figure S8 Sensitivity analysis of 5-year calibration performance of HCC prediction models in meta-analysis

**Content 15** Figure S9 Sensitivity analysis of 10-year calibration performance of HCC prediction models in meta-analysis

**Content 16** Figure S10 Sensitivity analysis of 5-year NPV prediction of HCC prediction models in meta-analysis

**Content 17** Figure S11 Sensitivity analysis of the low-risk population classified by the HCC prediction models in meta-analysis

**Content 18** Table S6 The pair-wise comparison of low-risk prediction between HCC-RESCUE and other models within the same investigations

1. Search strategy in each database

1.1 PubMed search srategy（540）

#1 Carcinoma, Hepatocellular[Mesh] 94450

#2 (((((((((((((((((Carcinomas, Hepatocellular[Title/Abstract]) OR (Hepatocellular Carcinomas[Title/Abstract])) OR (Liver Cell Carcinoma, Adult[Title/Abstract])) OR (Liver Cancer, Adult[Title/Abstract])) OR (Adult Liver Cancer[Title/Abstract])) OR (Adult Liver Cancers[Title/Abstract])) OR (Cancer, Adult Liver[Title/Abstract])) OR (Cancers, Adult Liver[Title/Abstract])) OR (Liver Cancers, Adult[Title/Abstract])) OR (Liver Cell Carcinoma[Title/Abstract])) OR (Carcinoma, Liver Cell[Title/Abstract])) OR (Carcinomas, Liver Cell[Title/Abstract])) OR (Cell Carcinoma, Liver[Title/Abstract])) OR (Cell Carcinomas, Liver[Title/Abstract])) OR (Liver Cell Carcinomas[Title/Abstract])) OR (Hepatocellular Carcinoma[Title/Abstract])) OR (Hepatoma[Title/Abstract])) OR (Hepatomas[Title/Abstract]) 132020

#3 #1 OR #2 149048

#4 Hepatitis B, Chronic[MeSH] 17302

#5 ((Chronic Hepatitis B Virus Infection[Title/Abstract]) OR (Chronic Hepatitis B[Title/Abstract])) OR (Hepatitis B Virus Infection, Chronic[Title/Abstract]) 18350

#6 #4 OR #5 24589

#7 ((((((((((((Predict* model*[Title/Abstract]) OR (predict* ind*[Title/Abstract])) OR (prediction rule*[Title/Abstract])) OR (risk score*[Title/Abstract])) OR (risk prediction model*[Title/Abstract])) OR (risk prediction score*[Title/Abstract])) OR (Validat*[Title/Abstract])) OR (ROC Curve[Title/Abstract])) OR (c-statistic[Title/Abstract])) OR (c statistic[Title/Abstract])) OR (Area under the curve[Title/Abstract])) OR (AUC[Title/Abstract])) OR (AUROC[Title/Abstract]) 772840

#8 (((((((((aMAP[Title/Abstract]) OR (REAL-B[Title/Abstract])) OR (CAGE-B[Title/Abstract])) OR (SAGE-B[Title/Abstract])) OR (CAMD[Title/Abstract])) OR (HCC-RESCUE[Title/Abstract])) OR (PAGE-B[Title/Abstract])) OR (mPAGE-B[Title/Abstract])) OR (mREACH-B[Title/Abstract])) OR (AASL-HCC[Title/Abstract]) 427

#9 #7 OR #8 773192

#10 #3 AND #6 AND #9 540

1.2 EMbase search strategy（620）

#1 'liver cell carcinoma'/exp 183961

#2 'carcinoma, hepatocellular':ab,ti OR 'carcinomas, hepatocellular':ab,ti OR 'hepatocellular carcinomas':ab,ti OR 'liver cell carcinoma, adult':ab,ti OR 'liver cancer, adult':ab,ti OR 'adult liver cancer':ab,ti OR 'adult liver cancers':ab,ti OR 'cancer, adult liver':ab,ti OR 'cancers, adult liver':ab,ti OR 'liver cancers, adult':ab,ti OR 'carcinoma, liver cell':ab,ti OR 'carcinomas, liver cell':ab,ti OR 'cell carcinoma, liver':ab,ti OR 'cell carcinomas, liver':ab,ti OR 'liver cell carcinomas':ab,ti OR 'hepatocellular carcinoma':ab,ti OR 'hepatoma':ab,ti OR 'hepatomas':ab,ti 177916

#3 #1 OR #2 222819

#4 'chronic hepatitis b'/exp 14974

#5 'chronic hepatitis b virus infection':ab,ti OR 'hepatitis b virus infection, chronic':ab,ti OR 'hepatitis b, chronic':ab,ti 2609

#6 #4 OR #5 16812

#7 'predict* model*':ab,ti OR 'predict* ind*':ab,ti OR 'prediction rule*':ab,ti OR 'risk score*':ab,ti OR 'risk prediction model*':ab,ti OR 'risk prediction score*':ab,ti OR validat*:ab,ti OR 'roc curve':ab,ti OR 'c statistic':ab,ti OR 'area under the curve':ab,ti OR auc:ab,ti OR auroc:ab,ti 1177942

#8 amap:ab,ti OR 'real b':ab,ti OR 'cage b':ab,ti OR 'sage b':ab,ti OR camd:ab,ti OR 'hcc rescue':ab,ti OR 'page b':ab,ti OR 'mpage b':ab,ti OR 'mreach b':ab,ti OR 'aasl hcc':ab,ti 612

#9 #7 OR #8 1178393

#10 #3 AND #6 AND #9 620

1.3 Cochrane search strategy (117)

#1 MeSH descriptor: [Carcinoma, Hepatocellular] explode all trees 1907

#2 MeSH descriptor: [Hepatitis B, Chronic] explode all trees 1332

#3 (Liver*):ti,ab,kw (Word variations have been searched) 55143

#4 (neoplas*):ti,ab,kw (Word variations have been searched) 88687

#5 (cancer):ti,ab,kw (Word variations have been searched) 177809

#6 (carcinoma*):ti,ab,kw (Word variations have been searched) 43968

#7 (tumor*):ti,ab,kw (Word variations have been searched) 70943

#8 (malignan*):ti,ab,kw (Word variations have been searched) 28596

#9 #3 AND (#4 OR #5 OR #6 OR #7 OR #8) 16790

#10 #1 OR #9 17169

#11 (Chronic Hepatitis B Virus Infection):ti,ab,kw (Word variations have been searched) 1651

#12 (Chronic Hepatitis B):ti,ab,kw (Word variations have been searched) 6567

#13 (Hepatitis B Virus Infection, Chronic):ti,ab,kw (Word variations have been searched) 1651

#14 #2 OR #11 OR #12 OR #13 6567

#15 (predict* model*):ti,ab,kw (Word variations have been searched) 28464

#16 (predict* ind*):ti,ab,kw (Word variations have been searched) 58597

#17 (prediction rule*):ti,ab,kw (Word variations have been searched) 1093

#18 (risk score*):ti,ab,kw (Word variations have been searched) 46041

#19 (risk prediction model*):ti,ab,kw (Word variations have been searched) 9079

#20 (risk prediction score*):ti,ab,kw (Word variations have been searched) 6886

#21 (validat*):ti,ab,kw (Word variations have been searched) 42962

#22 (roc curve):ti,ab,kw (Word variations have been searched) 3233

#23 (c-statistic):ti,ab,kw (Word variations have been searched) 738

#24 (c statistic):ti,ab,kw (Word variations have been searched) 23996

#25 (area under the curve):ti,ab,kw (Word variations have been searched) 32485

#26 (AUC):ti,ab,kw (Word variations have been searched) 21298

#27 (AUROC):ti,ab,kw (Word variations have been searched) 405

#28 #15 OR #16 OR #17 OR #18 OR #19 OR #20 OR #21 OR #22 OR #23 OR #24 OR #25 OR #26 OR #27 193479

#29 (aMAP):ti,ab,kw (Word variations have been searched) 23

#30 (REAL-B):ti,ab,kw (Word variations have been searched) 4

#31 (CAGE-B):ti,ab,kw (Word variations have been searched) 0

#32 (SAGE-B):ti,ab,kw (Word variations have been searched) 0

#33 (CAMD):ti,ab,kw (Word variations have been searched) 6

#34 (HCC-RESCUE):ti,ab,kw (Word variations have been searched) 0

#35 (PAGE-B):ti,ab,kw (Word variations have been searched) 2

#36 (mPAGE-B):ti,ab,kw (Word variations have been searched) 0

#37 (mREACH-B):ti,ab,kw (Word variations have been searched) 0

#38 (AASL-HCC):ti,ab,kw (Word variations have been searched) 0

#39 #29 OR #30 OR #31 OR #32 OR #33 OR #34 OR #35 OR #36 OR #37 OR #38 34

#40 #28 OR #39 193492

#41 #10 AND #14 AND #40 117 (Trials 117 + Cochrane reviews 7)

1.4 Web of Science search strategy (3097)

#1 TS=('Carcinoma, Hepatocellular' OR 'carcinoma, hepatocellular' OR 'carcinomas, hepatocellular' OR 'hepatocellular carcinomas' OR 'liver cell carcinoma, adult' OR 'liver cancer, adult' OR 'adult liver cancer' OR 'adult liver cancers' OR 'cancer, adult liver' OR 'cancers, adult liver' OR 'liver cancers, adult' OR 'carcinoma, liver cell' OR 'carcinomas, liver cell' OR 'cell carcinoma, liver' OR 'cell carcinomas, liver' OR 'liver cell carcinomas' OR 'hepatocellular carcinoma' OR 'hepatoma' OR 'hepatomas') 357156

#2 TS=('Hepatitis B, Chronic' OR 'Chronic Hepatitis B Virus Infection' OR 'Chronic Hepatitis B' OR 'Hepatitis B Virus Infection, Chronic') 75740

#3 TS=('predict* model*' OR 'predict* ind*' OR 'prediction rule*'i OR 'risk score*' OR 'risk prediction model*' OR 'risk prediction score*' OR validat* OR 'roc curve' OR 'c-statistic' OR 'c stastic' OR 'area under the curve' OR auc OR auroc) 5254279

#4 TS=(aMAP OR REAL-B OR CAGE-B OR SAGE-B OR CAMD OR HCC-RESCUE OR PAGE-B OR mPAGE-B OR mREACH-B OR AASL-HCC) 1521

#5 #3 OR #4 5255462

#6 #1 AND #2 AND #5 3097

Table S1 Risk of bias assessment of included studies according to PROBAST

| Study | Type | Participants | | Predictors | | | Outcome | | | | | | Analysis | | | | | | | | |
| --- | --- | --- | --- | --- | --- | --- | --- | --- | --- | --- | --- | --- | --- | --- | --- | --- | --- | --- | --- | --- | --- |
|  |  | Q1 | Q2 | Q3 | Q4 | Q5 | Q6 | Q7 | Q8 | Q9 | Q10 | Q11 | Q12 | Q13 | Q14 | Q15 | Q16 | Q17 | Q18 | Q19 | Q20 |
| Lee, 2014 | D | Y | Y | Y | Y | Y | NI | PY | Y | NI | Y | Y | N | Y | Y | Y | Y | Y | N | NI | NI |
| Papatheodoridis, 2016 | D+V | Y | Y | Y | Y | Y | Y | Y | Y | Y | Y | Y | Y | Y | Y | Y | Y | Y | Y | Y | Y |
| Chen, 2017 | V | Y | Y | Y | Y | Y | Y | Y | Y | Y | Y | Y | Y | N | Y | Y | NA | Y | N | NA | NA |
| Kim, 2017 | V | Y | Y | Y | Y | Y | Y | Y | Y | Y | Y | Y | Y | Y | Y | Y | NA | Y | N | NA | NA |
| Sohn, 2017 | D+V | Y | Y | Y | Y | Y | Y | Y | Y | Y | Y | Y | Y | Y | Y | Y | PY | Y | N | Y | Y |
| Hsu, 2018 | D+V | Y | Y | Y | Y | Y | Y | Y | Y | Y | Y | Y | Y | Y | Y | Y | PY | Y | Y | Y | Y |
| Kim, 2018 | D+V | Y | Y | Y | Y | Y | Y | Y | Y | Y | Y | Y | Y | Y | Y | Y | PY | Y | Y | Y | N |
| Yu, 2019 | D+V | Y | Y | Y | Y | Y | Y | Y | Y | Y | Y | Y | Y | Y | Y | Y | Y | Y | Y | Y | Y |
| Fan, 2020 | D+V | Y | Y | Y | Y | Y | Y | Y | Y | Y | Y | Y | N | Y | N | Y | Y | Y | Y | Y | Y |
| Kim, 2020 | V | Y | Y | Y | Y | Y | Y | Y | Y | Y | Y | Y | Y | Y | Y | Y | NA | Y | Y | NA | NA |
| Kirino, 2020 | V | Y | PY | Y | Y | Y | Y | Y | Y | Y | Y | Y | N | Y | Y | Y | NA | Y | Y | NA | NA |
| Papatheodoridis, 2020 | D | Y | Y | Y | Y | Y | Y | Y | Y | Y | Y | Y | Y | Y | Y | Y | Y | Y | Y | Y | Y |
| Yip, 2020 | V | Y | N | Y | Y | Y | PY | PY | PY | PY | PY | PY | Y | Y | Y | Y | NA | Y | Y | NA | NA |
| Ahn, 2021 | V | Y | PY | Y | Y | Y | Y | Y | Y | Y | Y | Y | Y | Y | Y | Y | NA | Y | N | NA | NA |
| Chang, 2021 | V | Y | Y | Y | Y | Y | Y | Y | Y | Y | Y | Y | Y | Y | Y | Y | NA | Y | Y | NA | NA |
| Chon, 2021 | V | Y | Y | Y | Y | Y | Y | Y | Y | Y | Y | Y | Y | Y | Y | Y | NA | Y | N | NA | NA |
| Gui, 2021 | V | Y | N | Y | Y | Y | Y | Y | Y | Y | Y | Y | Y | Y | Y | Y | NA | Y | Y | NA | NA |
| Güzelbulut, 2021 | V | Y | N | Y | Y | Y | Y | Y | Y | Y | Y | Y | N | Y | Y | Y | NA | Y | Y | NA | NA |
| Lee, 2021 | V | Y | PY | Y | Y | Y | Y | Y | Y | Y | Y | Y | Y | Y | Y | Y | NA | Y | N | NA | NA |
| Lim, 2021 | V | Y | Y | Y | Y | Y | Y | Y | Y | Y | Y | Y | Y | Y | Y | Y | NA | Y | Y | NA | NA |
| Papatheodoridis, 2021 | V | Y | Y | Y | Y | Y | Y | Y | Y | Y | Y | Y | Y | Y | Y | Y | NA | Y | Y | NA | NA |
| Chon, 2022 | V | Y | Y | Y | Y | Y | Y | Y | Y | Y | Y | Y | Y | Y | Y | Y | NA | Y | Y | NA | NA |
| Kim, 2022 | V | N | PY | Y | Y | Y | Y | Y | Y | Y | Y | Y | Y | Y | Y | Y | NA | Y | N | NA | NA |

D, model development; V, model validation; Y: yes; PY, probably yes; N, no; NA, not applicable; NI: no information

Table S2 Risk of bias and applicability analysis of included studies according to PROBAST

| Study | ROB | | | | Applicability | | | Overall | |
| --- | --- | --- | --- | --- | --- | --- | --- | --- | --- |
|  | Participants | Predictors | Outcome | Analysis | Participants | Predictors | Outcome | ROB | Applicability |
| Lee, 2014 | Low | Low | Unclear | High | Low | Low | Unclear | High | Unclear |
| Papatheodoridis, 2016 | Low | Low | Low | Low | Low | Low | Low | Low | Low |
| Chen, 2017 | Low | Low | Low | High | Low | Low | Low | High | Low |
| Kim, 2017 | Low | Low | Low | High | Low | Low | Low | High | Low |
| Sohn, 2017 | Low | Low | Low | High | Low | Low | Low | High | Low |
| Hsu, 2018 | Low | Low | Low | Low | Low | Low | Low | Low | Low |
| Kim, 2018 | Low | Low | Low | High | Low | Low | Low | High | Low |
| Yu, 2019 | Low | Low | Low | Low | Low | Low | Low | Low | Low |
| Fan, 2020 | Low | Low | Low | High | Low | Low | Low | High | Low |
| Kim, 2020 | Low | Low | Low | Low | Low | Low | Low | Low | Low |
| Kirino, 2020 | Low | Low | Low | High | Low | Low | Low | High | High |
| Papatheodoridis, 2020 | Low | Low | Low | Low | Low | Low | Low | Low | Low |
| Yip, 2020 | High | Low | Unclear | Low | High | Low | Unclear | High | High |
| Ahn, 2021 | Low | Low | Low | High | Low | Low | Low | High | Low |
| Chang, 2021 | Low | Low | Low | Low | Low | Low | Low | Low | Low |
| Chon, 2021 | Low | Low | Low | High | Low | Low | Low | High | Low |
| Gui, 2021 | High | Low | Low | Low | High | Low | Low | High | High |
| Güzelbulut, 2021 | High | Low | Low | High | High | Low | Low | High | High |
| Lee, 2021 | Low | Low | Low | High | Low | Low | Low | High | Low |
| Lim, 2021 | Low | Low | Low | Low | Low | Low | Low | Low | Low |
| Papatheodoridis, 2021 | Low | Low | Low | Low | Low | Low | Low | Low | Low |
| Chon, 2022 | Low | Low | Low | Low | Low | Low | Low | Low | Low |
| Kim, 2022 | High | Low | Low | High | High | Low | Low | High | High |


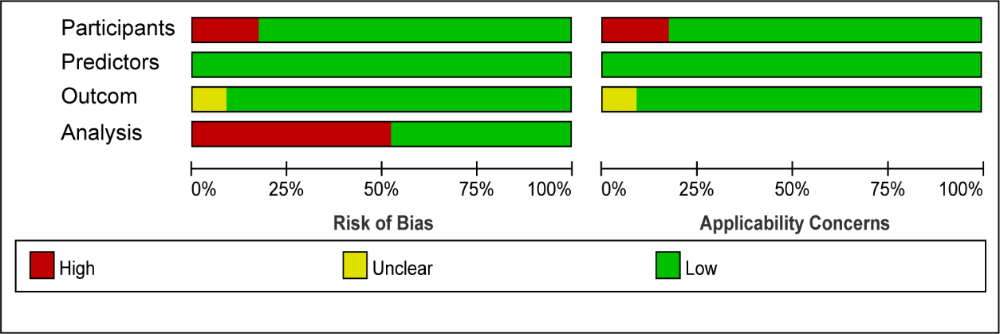


Figure S1 Risk of bias and applicability concerns graph: review authors' judgements about each domain presented as percentages across included studies


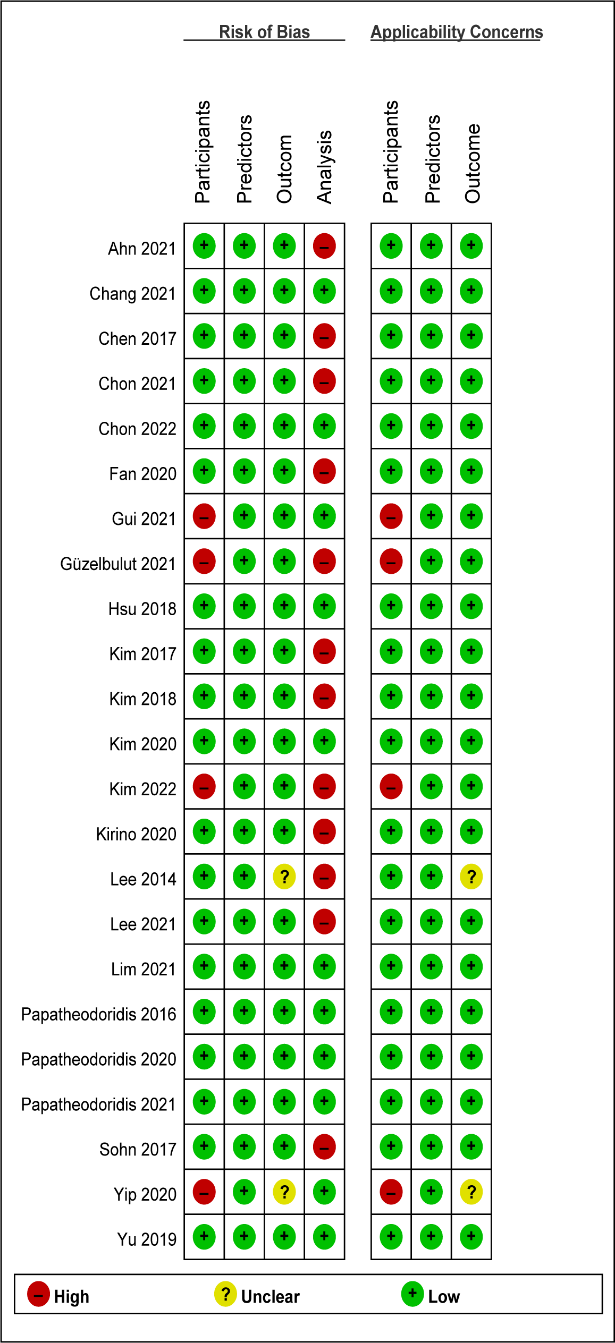


Figure S2 Risk of bias and applicability concerns summary: review authors' judgements about each domain for each included study


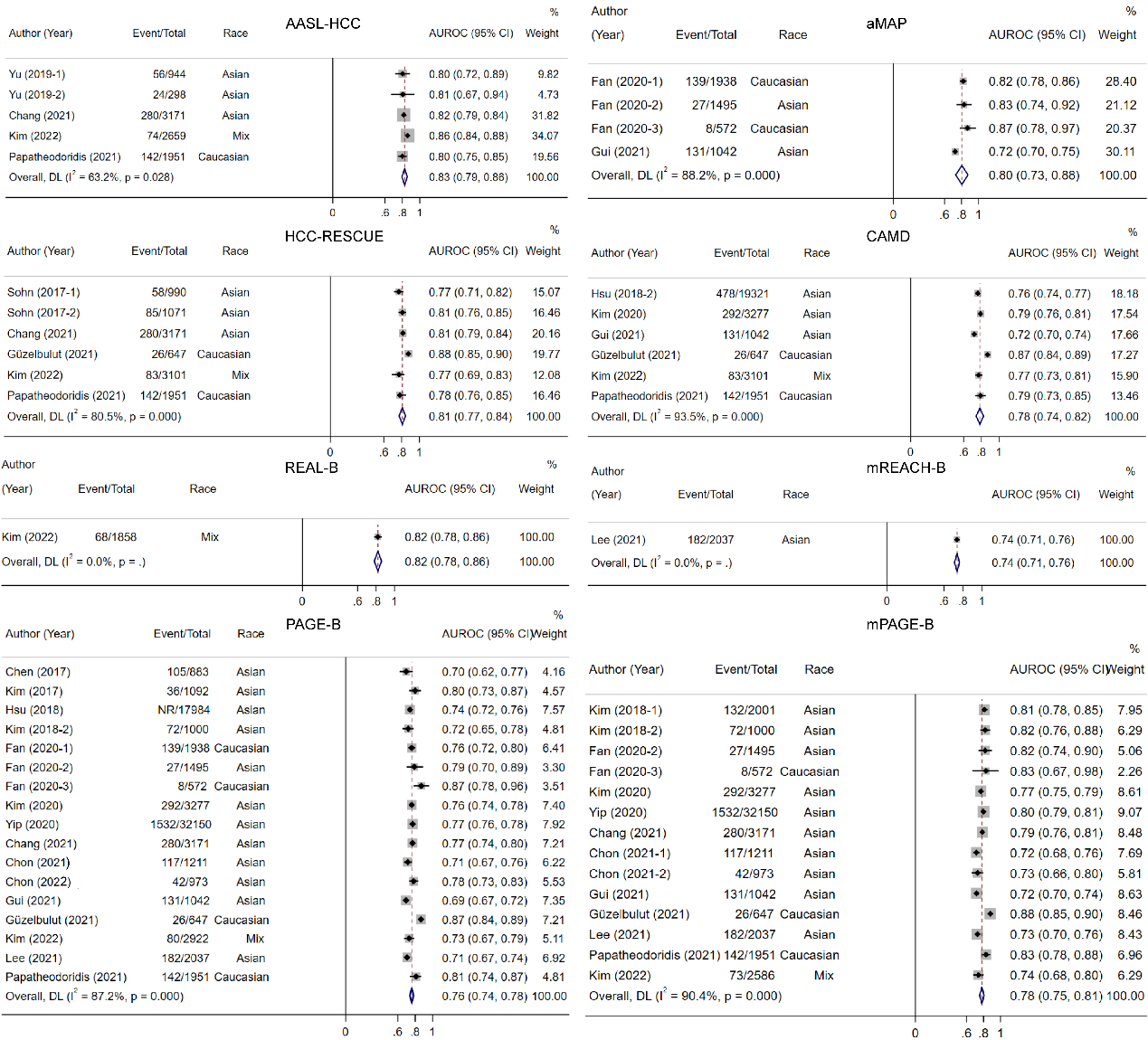


Figure S3 The 5-year discrimination performance of each HCC prediction models in meta-analysis

AUROC, area under the receiver operator characteristic curve; CI, confidence interval; HCC, hepatocellular carcinoma; mREACH-B, Modified Risk Estimation for Hepatocellular Carcinoma in Chronic Hepatitis B; PAGE-B, Platelet, Age, Gender and HBV; mPAGE-B, modified Platelet, Age, Gender and HBV; HCC-RESCUE, HCC-Risk Estimating Score in CHB patients Under Entecavir; CAMD, the Cirrhosis, Age, Male sex, and Diabetes Mellitus Score; AASL-HCC, Age, Albumin, Aex, Liver Cirrhosis-HCC scoring system; aMAP: the Age-Male-ALBI-Platelets Score; CAGE-B, Cirrhosis and Age Score; SAGE-B, Stiffness and Age Score; REAL-B, Real-world Effectiveness from the Asia Pacific Rim Liver Consortium for HBV

Table S3 The 3- and 10-year discrimination performance of HCC prediction models in meta-analysis

| Model | Time | Cohorts | AUROC | 95% CI | I^2^ | *P* |
| --- | --- | --- | --- | --- | --- | --- |
| mREACH-B | 3-year | N = 2 | 0.74 | 0.71, 0.78 | 7.2% | 0.299 |
| PAGE-B | 3-year | N = 8 | 0.74 | 0.72, 0.76 | 56% | 0.026 |
|  | 10-year | N = 4 | 0.77 | 0.70, 0.84 | 86.0% | < 0.001 |
| mPAGE-B | 3-year | N = 4 | 0.78 | 0.76, 0.80 | 30.8% | 0.227 |
|  | 10-year | N = 1 | 0.87 | 0.83, 0.89 | 0.0% | - |
| HCC-RESCUE | 3-year | N = 4 | 0.81 | 0.79, 0.82 | 0.0% | 0.717 |
|  | 10-year | N = 2 | 0.84 | 0.79, 0.89 | 68.7% | 0.074 |
| CAMD | 3-year | N = 3 | 0.79 | 0.73, 0.84 | 91.0% | < 0.001 |
|  | 10-year | N = 2 | 0.83 | 0.78, 0.87 | 57.4% | 0.125 |
| AASL-HCC | 3-year | N = 4 | 0.83 | 0.81, 0.86 | 0.0% | 0.405 |
|  | 10-year | N = 3 | 0.80 | 0.78, 0.83 | 0.0% | 0.809 |
| aMAP | 3-year | N = 1 | 0.72 | 0.70, 0.75 | 0.0% | - |
| CAGE-B | 10-year | N = 3 | 0.79 | 0.76, 0.83 | 0.0% | 0.876 |
| SAGE-B | 10-year | N = 3 | 0.76 | 0.70, 0.83 | 63.9% | 0.062 |
| REAL-B | 3-year | N = 1 | 0.84 | 0.78, 0.90 | 0.0% | - |

AUROC, area under the receiver operator characteristic curve; CI, confidence interval; HCC, hepatocellular carcinoma; mREACH-B, Modified Risk Estimation for Hepatocellular Carcinoma in Chronic Hepatitis B; PAGE-B, Platelet, Age, Gender and HBV; mPAGE-B, modified Platelet, Age, Gender and HBV; HCC-RESCUE, HCC-Risk Estimating Score in CHB patients Under Entecavir; CAMD, the Cirrhosis, Age, Male sex, and Diabetes Mellitus Score; AASL-HCC, Age, Albumin, Aex, Liver Cirrhosis-HCC scoring system; aMAP: the Age-Male-ALBI-Platelets Score; CAGE-B, Cirrhosis and Age Score; SAGE-B, Stiffness and Age Score; REAL-B, Real-world Effectiveness from the Asia Pacific Rim Liver Consortium for HBV


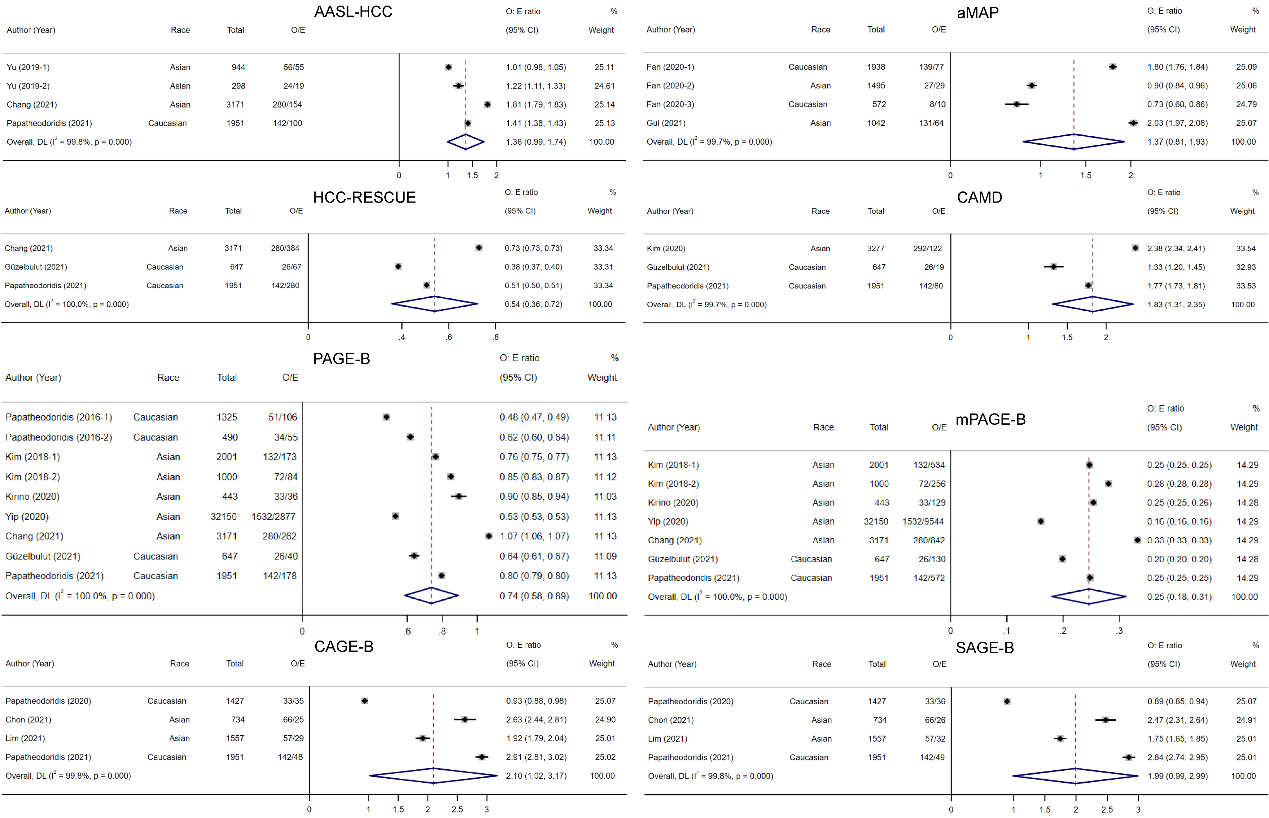


Figure S4 The 5- and 10-year calibration performance of each HCC prediction models in meta-analysis

CAGE-B and SAGE-B were evaluated for 10-year performance, while other models were evaluated for 5-year performance. CI, confidence interval; HCC, hepatocellular carcinoma; O:E ratio, observed events versus expected events ratio; PAGE-B, Platelet, Age, Gender and HBV; mPAGE-B, modified Platelet, Age, Gender and HBV; HCC-RESCUE, HCC-Risk Estimating Score in CHB patients Under Entecavir; CAMD, the Cirrhosis, Age, Male sex, and Diabetes Mellitus Score; AASL-HCC, Age, Albumin, Aex, Liver Cirrhosis-HCC scoring system; aMAP: the Age-Male-ALBI-Platelets Score; CAGE-B, Cirrhosis and Age Score; SAGE-B, Stiffness and Age Score

Table S4 The 3-, 5- and 10-year NPV prediction of HCC prediction models in meta-analysis

| Model | Time | Cohorts | NPV, % | 95% CI | I^2^ | *P* |
| --- | --- | --- | --- | --- | --- | --- |
| PAGE-B | 3-year | N = 4 | 99.1 | 97.7, 100.5 | 54.9% | 0.084 |
|  | 5-year | N = 9 | 99.7 | 99.4, 100.0 | 26.5% | 0.209 |
|  | 10-year | N = 1 | 99.6 | 98.4, 100.0 | 0.0% | - |
| mPAGE-B | 3-year | N = 3 | 99.7 | 99.4, 100.3 | 0.0% | 0.505 |
|  | 5-year | N = 4 | 99.8 | 99.4, 100.1 | 41.0% | 0.166 |
|  | 10-year | N = 1 | 99.7 | 98.8, 100.7 | 0.0% | - |
| HCC-RESCUE | 5-year | N = 1 | 100.0 | 99.6, 100.5 | 0.0% | - |
|  | 10-year | N = 1 | 99.7 | 99.0, 100.4 | 0.0% | - |
| CAMD | 3-year | N = 3 | 99.8 | 99.5, 100.0 | 87.0% | < 0.001 |
|  | 5-year | N = 3 | 99.7 | 99.3, 100.1 | 73.9% | 0.022 |
|  | 10-year | N = 1 | 99.7 | 98.9, 100.5 | 0.0% | - |
| AASL-HCC | 5-year | N = 3 | 100.0 | 99.5, 100.5 | 0.0% | 1.000 |
| aMAP | 3-year | N = 1 | 98.3 | 96.3, 100.3 | 0.0% | - |
|  | 5-year | N = 4 | 99.6 | 99.2, 100.0 | 16.4% | - |
| CAGE-B | 10-year | N = 1 | 100.0 | 99.4, 100.7 | 0.0% | - |
| SAGE-B | 10-year | N = 1 | 100.0 | 99.4, 100.6 | 0.0% | - |
| REAL-B | 3-year | N = 1 | 100.0 | 99.5, 100.5 | 0.0% | - |
|  | 5-year | N = 1 | 100.0 | 99.5, 100.5 | 0.0% | - |

NPV, negative predictive value; CI, confidence interval; HCC, hepatocellular carcinoma; mREACH-B, Modified Risk Estimation for Hepatocellular Carcinoma in Chronic Hepatitis B; PAGE-B, Platelet, Age, Gender and HBV; mPAGE-B, modified Platelet, Age, Gender and HBV; HCC-RESCUE, HCC-Risk Estimating Score in CHB patients Under Entecavir; CAMD, the Cirrhosis, Age, Male sex, and Diabetes Mellitus Score; AASL-HCC, Age, Albumin, Aex, Liver Cirrhosis-HCC scoring system; aMAP: the Age-Male-ALBI-Platelets Score; CAGE-B, Cirrhosis and Age Score; SAGE-B, Stiffness and Age Score; REAL-B, Real-world Effectiveness from the Asia Pacific Rim Liver Consortium for HBV

Table S5 Subgroup analysis of model discrimination (A) and calibration (B) performance

1. **discrimination**

| Model | Time | Cohorts | Subgroup | AUROC | 95% CI | I^2^ | *P* |
| --- | --- | --- | --- | --- | --- | --- | --- |
| PAGE-B | 5-year | N = 5 | Cirrhosis | 0.69 | 0.63, 0.74 | 83.7% | < 0.001 |
|  |  | N = 3 | Non-cirrhosis | 0.83 | 0.68, 0.99 | 93.8% | < 0.001 |
|  |  | N = 4 | Caucasian | 0.82 | 0.76, 0.89 | 84.7% | < 0.001 |
|  |  | N = 12 | Asian | 0.74 | 0.72, 0.76 | 79.6% | < 0.001 |
|  |  | N = 1 | Mix | 0.73 | 0.67, 0.79 | 0.0% | - |
| mPAGE-B | 5-year | N = 4 | Cirrhosis | 0.69 | 0.63, 0.76 | 87.2% | < 0.001 |
|  |  | N = 2 | Non-cirrhosis | 0.89 | 0.74, 1.04 | 89.2% | 0.002 |
|  |  | N = 3 | Caucasian | 0.86 | 0.82, 0.90 | 38.8% | 0.195 |
|  |  | N = 10 | Asian | 0.77 | 0.74, 0.79 | 88.3% | < 0.001 |
|  |  | N = 1 | Mix | 0.74 | 0.68, 0.80 | 0.0% | - |
| HCC-RESCUE | 5-year | N = 2 | Caucasian | 0.83 | 0.74, 0.92 | 92.1% | < 0.001 |
|  |  | N = 3 | Asian | 0.80 | 0.78, 0.83 | 18.5% | 0.293 |
|  |  | N = 1 | Mix | 0.77 | 0.70, 0.84 | 0.0% | - |
| CAMD | 5-year | N = 2 | Caucasian | 0.83 | 0.76, 0.91 | 82.4% | 0.017 |
|  |  | N = 3 | Asian | 0.76 | 0.72, 0.79 | 89.1% | < 0.001 |
|  |  | N = 1 | Mix | 0.77 | 0.73, 0.81 | 0.0% | - |
| AASL-HCC | 5-year | N = 1 | Caucasian | 0.80 | 0.75, 0.85 | 0.0% | - |
|  |  | N = 3 | Asian | 0.81 | 0.79, 0.84 | 0.0% | 0.944 |
|  |  | N = 1 | Mix | 0.86 | 0.84, 0.88 | 0.0% | - |
| aMAP | 5-year | N = 4 | Cirrhosis | 0.72 | 0.70, 0.74 | 0.0% | 0.537 |
|  |  | N = 3 | Non-cirrhosis | 0.88 | 0.77, 0.98 | 88.1% | < 0.001 |
|  |  | N = 2 | Caucasian | 0.83 | 0.79, 0.86 | 0.0% | 0.342 |
|  |  | N = 2 | Asian | 0.77 | 0.67, 0.87 | 80.0% | 0.025 |
| CAGE-B | 10-year | N = 1 | Caucasian | 0.79 | 0.66,0.93 | 0.0% | - |
|  |  | N = 2 | Asian | 0.79 | 0.76, 0.83 | 0.0% | 0.608 |
| SAGE-B | 10-year | N = 1 | Caucasian | 0.76 | 0.65, 0.91 | 0.0% | - |
|  |  | N = 2 | Asian | 0.76 | 0.67, 0.85 | 82.0% | 0.019 |

1. **Calibration**

| Model | Time | Cohort | Subgroup | O:E ratio | 95% CI | I^2^ | *P* |
| --- | --- | --- | --- | --- | --- | --- | --- |
| PAGE-B | 5-year | N = 4 | Caucasian | 0.63 | 0.44, 0.82 | 99.9% | < 0.001 |
|  |  | N = 5 | Asian | 0.82 | 0.56, 1.08 | 100.0% | < 0.001 |
| mPAGE-B | 5-year | N = 2 | Caucasian | 0.22 | 0.18, 0.27 | 99.9% | < 0.001 |
|  |  | N = 5 | Asian | 0.25 | 0.17, 0.34 | 100.0% | < 0.001 |
| HCC-RESCUE | 5-year | N = 2 | Caucasian | 0.45 | 0.33, 0.56 | 99.8% | < 0.001 |
|  |  | N = 1 | Asian | 0.73 | 0.73, 0.73 | 0.0% | - |
| CAMD | 5-year | N = 2 | Caucasian | 1.55 | 1.12, 1.99 | 97.7% | < 0.001 |
|  |  | N = 1 | Asian | 2.38 | 2.34, 2.41 | 0.0% | - |
| AASL-HCC | 5-year | N = 1 | Caucasian | 1.41 | 1.38, 1.43 | 0.0% | - |
|  |  | N = 3 | Asian | 1.35 | 0.73, 1.97 | 99.9% | < 0.001 |
| aMAP | 5-year | N = 2 | Caucasian | 1.27 | 0.22, 2.31 | 99.6% | < 0.001 |
|  |  | N = 2 | Asian | 1.46 | 0.36, 2.57 | 99.9% | < 0.001 |
| CAGE-B | 10-year | N = 2 | Caucasian | 1.92 | -0.02, 3.87 | 99.9% | < 0.001 |
|  |  | N = 2 | Asian | 2.27 | 1.57, 2.96 | 97.4% | < 0.001 |
| SAGE-B | 10-year | N = 2 | Caucasian | 1.87 | -0.04, 3.78 | 99.9% | < 0.001 |
|  |  | N = 2 | Asian | 2.11 | 1.39, 2.82 | 98.2% | < 0.001 |

AUROC, area under the receiver operator characteristic curve; CI, confidence interval; O:E ratio, observed events versus expected events ratio; PAGE-B, Platelet, Age, Gender and HBV; mPAGE-B, modified Platelet, Age, Gender and HBV; HCC-RESCUE, HCC-Risk Estimating Score in CHB patients Under Entecavir; CAMD, the Cirrhosis, Age, Male sex, and Diabetes Mellitus Score; AASL-HCC, Age, Albumin, Aex, Liver Cirrhosis-HCC scoring system; aMAP: the Age-Male-ALBI-Platelets Score; CAGE-B, Cirrhosis and Age Score; SAGE-B, Stiffness and Age Score;


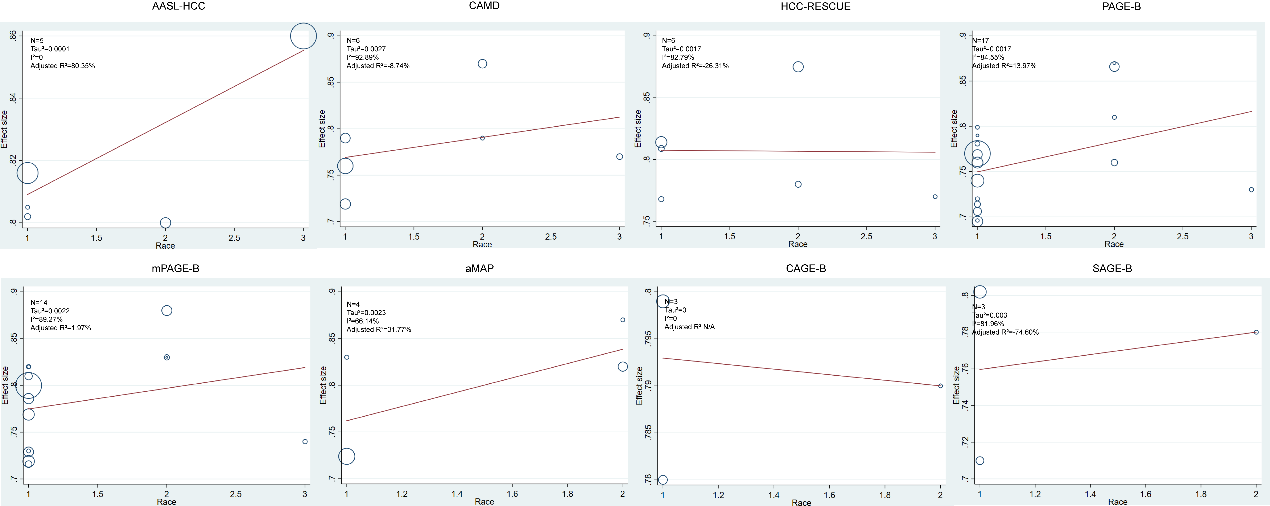


(A)


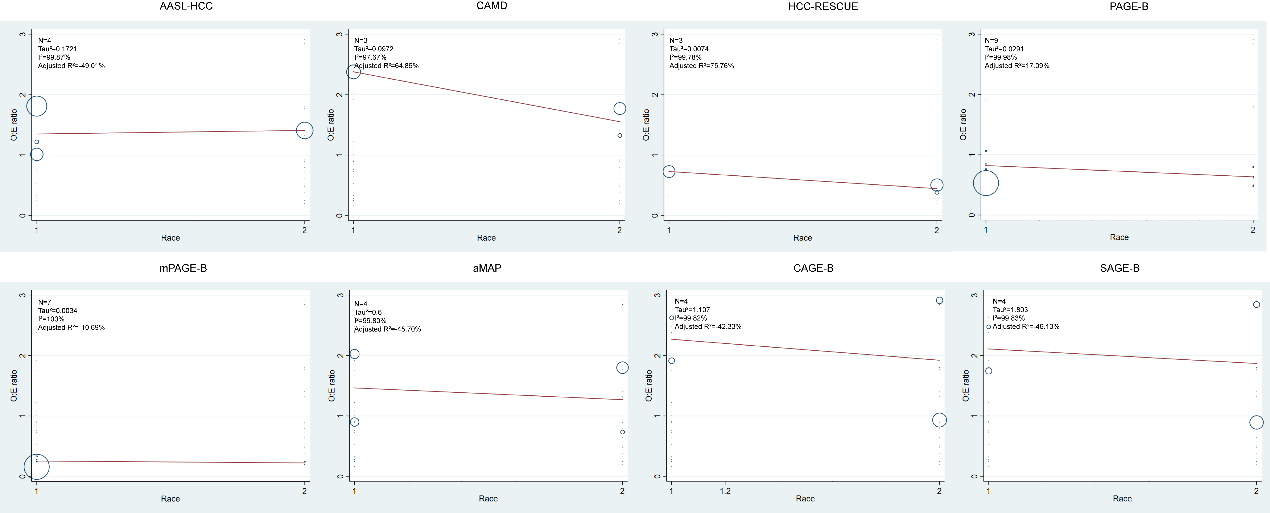


(B)

Figure S5 Meta-regression analysis of model discrimination (A) and calibration (B) performance

The 5-year discrimination and calibration performance were analyzed except for CAGE-B and SAGE-B (10-year). Adjusted by the Hartung-Knapp adjustment. PAGE-B, Platelet, Age, Gender and HBV; mPAGE-B, modified Platelet, Age, Gender and HBV; HCC-RESCUE, HCC-Risk Estimating Score in CHB patients Under Entecavir; CAMD, the Cirrhosis, Age, Male sex, and Diabetes Mellitus Score; AASL-HCC, Age, Albumin, Aex, Liver Cirrhosis-HCC scoring system; aMAP: the Age-Male-ALBI-Platelets Score; CAGE-B, Cirrhosis and Age Score; SAGE-B, Stiffness and Age Score


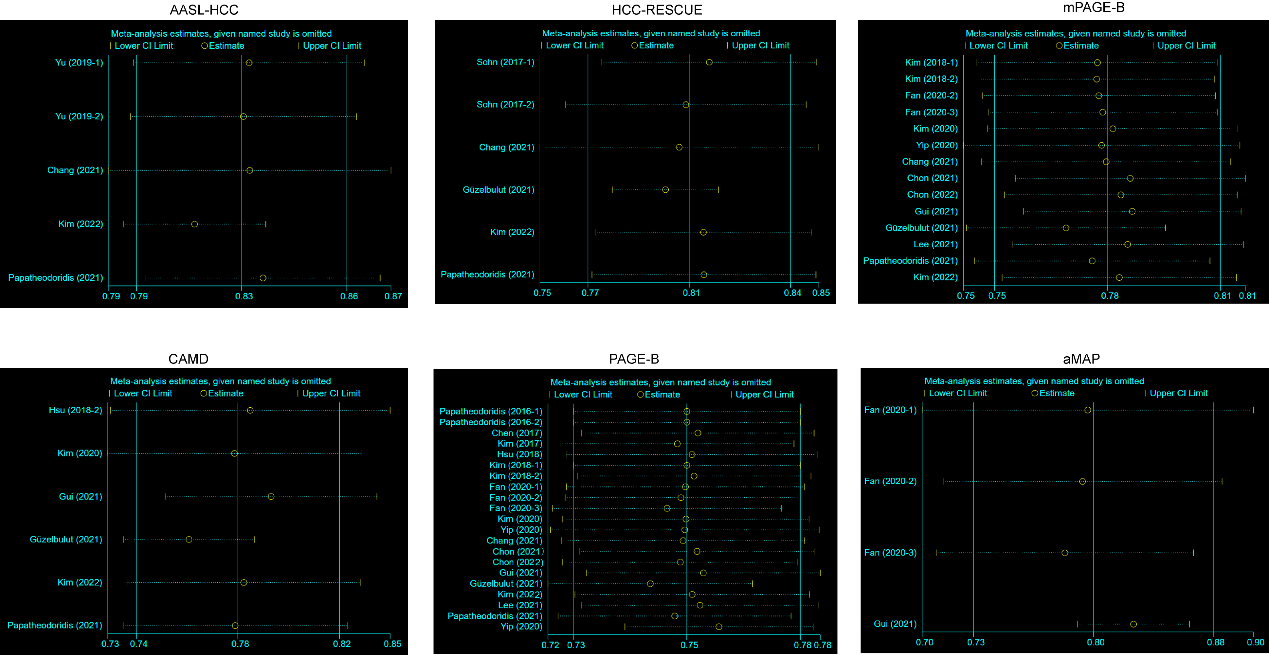


Figure S6 Sensitivity analysis of 5-year discrimination performance of HCC prediction models in meta-analysis

CI, confidence interval; PAGE-B, Platelet, Age, Gender and HBV; mPAGE-B, modified Platelet, Age, Gender and HBV; HCC-RESCUE, HCC-Risk Estimating Score in CHB patients Under Entecavir; CAMD, the Cirrhosis, Age, Male sex, and Diabetes Mellitus Score; AASL-HCC, Age, Albumin, Aex, Liver Cirrhosis-HCC scoring system; aMAP: the Age-Male-ALBI-Platelets Score;


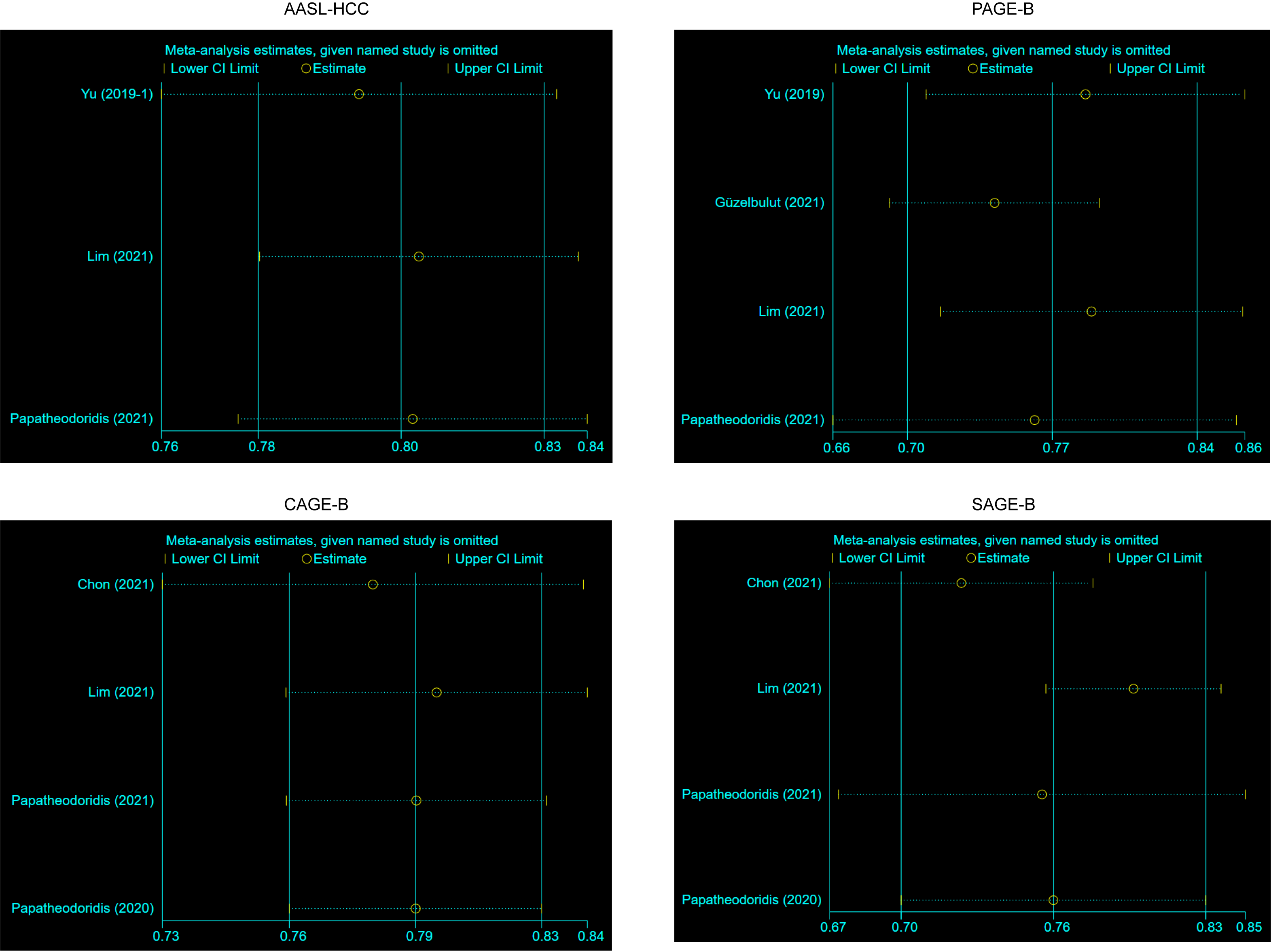


Figure S7 Sensitivity analysis of 10-year discrimination performance of HCC prediction models in meta-analysis

CI, confidence interval; PAGE-B, Platelet, Age, Gender and HBV; AASL-HCC, Age, Albumin, Aex, Liver Cirrhosis-HCC scoring system; CAGE-B, Cirrhosis and Age Score; SAGE-B, Stiffness and Age Score


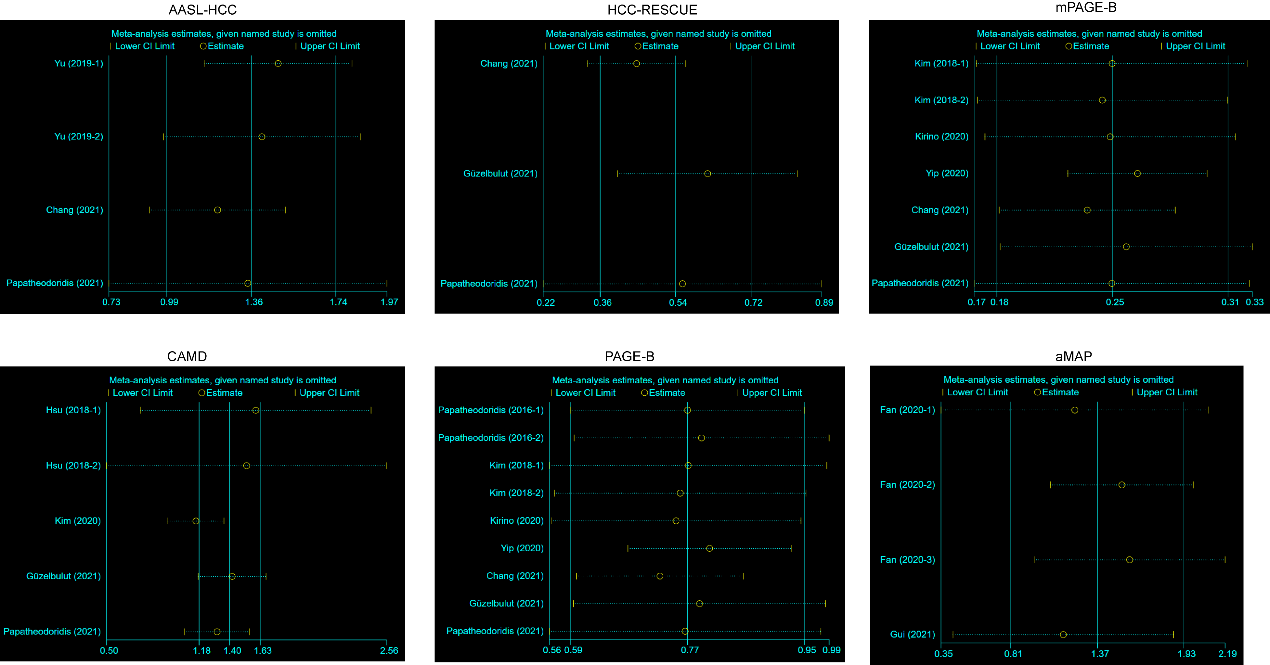


Figure S8 Sensitivity analysis of 5-year calibration performance of HCC prediction models in meta-analysis

CI, confidence interval; PAGE-B, Platelet, Age, Gender and HBV; mPAGE-B, modified Platelet, Age, Gender and HBV; HCC-RESCUE, HCC-Risk Estimating Score in CHB patients Under Entecavir; CAMD, the Cirrhosis, Age, Male sex, and Diabetes Mellitus Score; AASL-HCC, Age, Albumin, Aex, Liver Cirrhosis-HCC scoring system; aMAP: the Age-Male-ALBI-Platelets Score


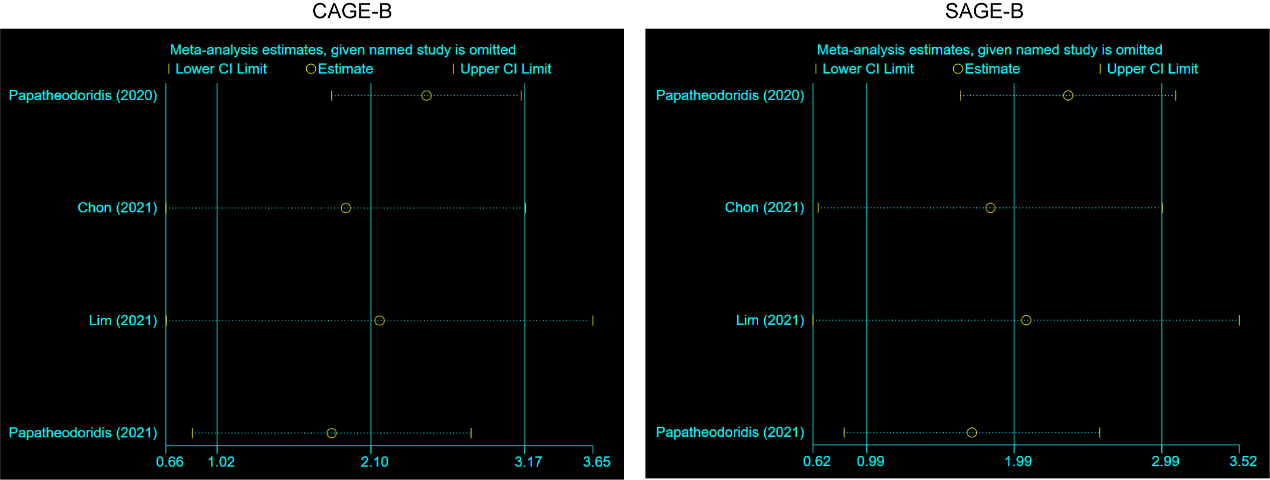


Figure S9 Sensitivity analysis of 10-year calibration performance of HCC prediction models in meta-analysis

CI, confidence interval; CAGE-B, Cirrhosis and Age Score; SAGE-B, Stiffness and Age Score


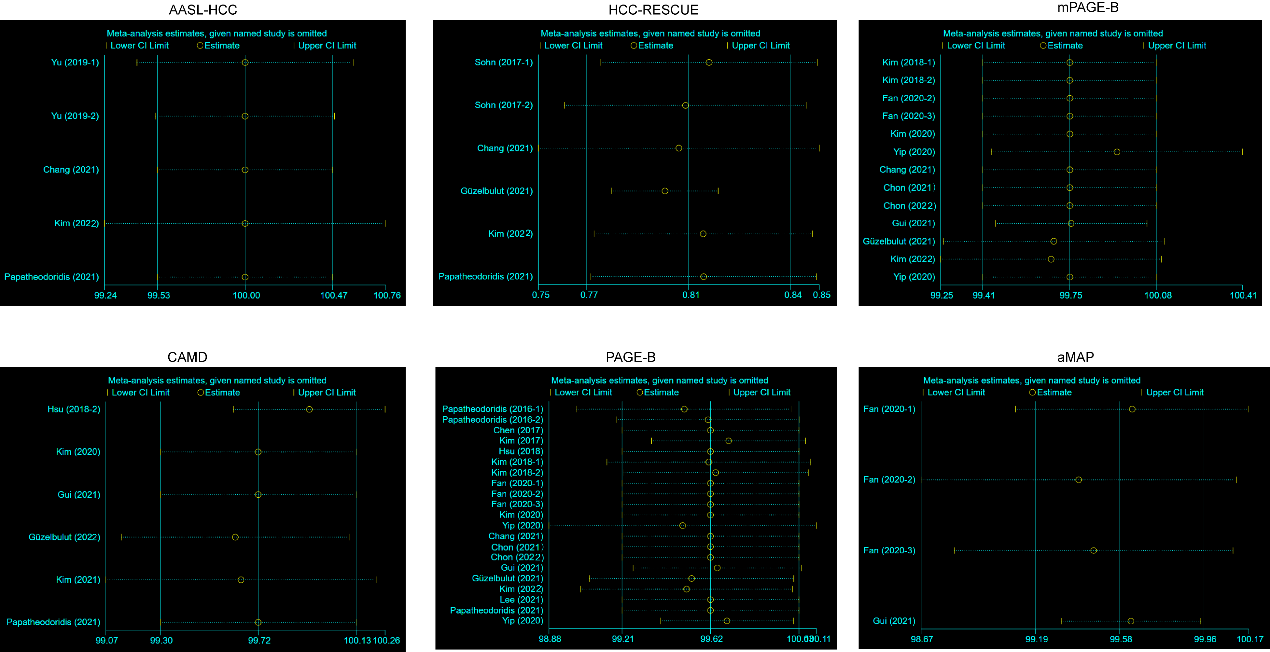


Figure S10 Sensitivity analysis of 5-year NPV prediction of HCC prediction models in meta-analysis

CI, confidence interval; PAGE-B, Platelet, Age, Gender and HBV; mPAGE-B, modified Platelet, Age, Gender and HBV; HCC-RESCUE, HCC-Risk Estimating Score in CHB patients Under Entecavir; CAMD, the Cirrhosis, Age, Male sex, and Diabetes Mellitus Score; AASL-HCC, Age, Albumin, Aex, Liver Cirrhosis-HCC scoring system; aMAP: the Age-Male-ALBI-Platelets Score


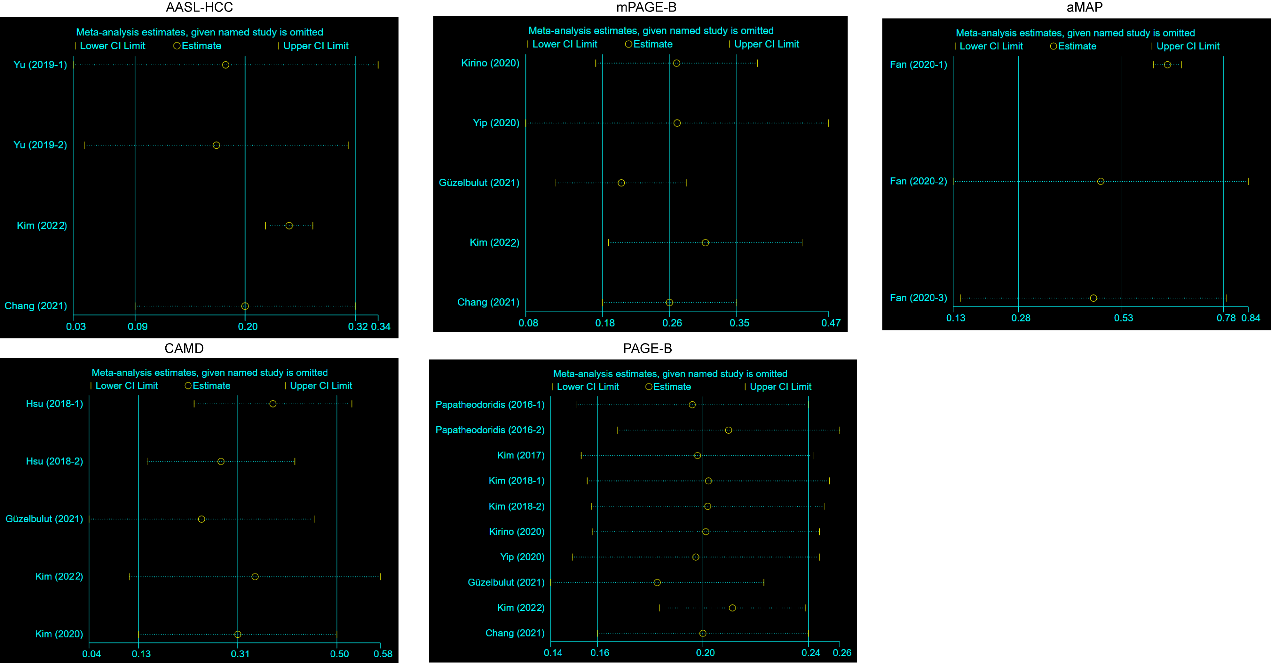


Figure S11 Sensitivity analysis of the low-risk population classified by the HCC prediction models in meta-analysis

CI, confidence interval; PAGE-B, Platelet, Age, Gender and HBV; mPAGE-B, modified Platelet, Age, Gender and HBV; CAMD, the Cirrhosis, Age, Male sex, and Diabetes Mellitus Score; AASL-HCC, Age, Albumin, Aex, Liver Cirrhosis-HCC scoring system; aMAP: the Age-Male-ALBI-Platelets Score

**Table S6** The pair-wise comparison of low-risk prediction between HCC-RESCUE and other models within the same investigations.

| Model | Sample size | Low-risk proportion, % | 95% CI | I^2^ | *P* |
| --- | --- | --- | --- | --- | --- |
| PAGE-B | 3818 (N = 2) | 23.2 | 21.9, 24.5 | 0.0% | - |
| mPAGE-B | 3818 (N = 2) | 30.0 | 28.6, 31.4 | 0.0% | - |
| HCC-RESCUE | 3818 (N = 2) | 52.4 | 50.8, 54.0 | 0.0% | - |

CI, confidence interval; PAGE-B, Platelet, Age, Gender and HBV; mPAGE-B, modified Platelet, Age, Gender and HBV; HCC-RESCUE, HCC-Risk Estimating Score in CHB patients Under Entecavir
